# Are Self-transcendent Emotions One Big Family? An Empirical Taxonomy of Positive Self-transcendent Emotion Labels

**DOI:** 10.1007/s42761-023-00194-1

**Published:** 2023-06-19

**Authors:** Angela Gaia F. Abatista, Florian Cova

**Affiliations:** 1 Swiss Center for Affective Sciences, University of Geneva, Campus Biotech, Chemin Des Mines 9, 1202 Geneva, Switzerland; 2 Department of Philosophy, University of Geneva, Rue de Candolle 2, 1205 Geneva, Switzerland; 3 Psychology Department, Université Grenoble Alpes, 1251 Avenue Centrale, 38400 Saint-Martin-d’Hères, France; 4 Geneva Centre for Philanthropy, University of Geneva, Rue du Général Dufour, 24, 1211 Geneva, Switzerland

**Keywords:** Self-transcendent emotions, Positive emotions, Taxonomy, Emotion families, Subjective feeling, Data-driven

## Abstract

In the past decade, there has been a growing amount of research on so-called self-transcendent emotions, mainly in the domain of positive emotions. However, most candidate self-transcendent emotions (e.g., Awe, Gratitude, Being Moved, Wonder) have been studied in isolation, leaving the commonalities and the differences of their phenomenology unknown. In the present paper, we sought to identify the phenomenological nature of main families of self-transcendent emotions. We drew on two large datasets (*N*_1_ = 3,113; *N*_2_ = 1,443) in which participants had to recall an emotional episode or to watch emotional videos and had to report their emotions through a list of 40 emotion labels. Participants were also presented with a large list of items probing their cognitive appraisals, bodily feelings, and action tendencies. Using a principal component analysis, we identified three main dimensions of positive emotions: hedonic, social, and epistemic states. Candidate self-transcendent emotions were distributed across two dimensions, suggesting that at least two main different families of self-transcendent emotions should be distinguished. Our results also allowed us to identify self-reported cognitive appraisals, bodily feelings, and action tendencies characteristic of each family.

**Supplementary Information:**

The online version contains supplementary material available at 10.1007/s42761-023-00194-1.

Fifteen years ago, psychologists interested in emotions regularly complained that positive emotions had received too little interest compared to negative emotions (Fredrickson, 2008). However, the last two decades witnessed the development of a rich literature differentiating positive emotions, an important part of which has focused on the so-called self-transcendent emotions (Stellar et al., 2017). As such, psychologists have investigated potential self-transcendent emotions as diverse as “Gratitude” (Emmons & McCullough, 2004), “Elevation” (Haidt, 2003a), “Awe” (Keltner & Haidt, 2003), “Admiration” (Onu et al., 2016), “Adoration” (Schindler et al., 2013), “Wonder” (Lamont, 2017), “Being Moved” (Cova & Deonna, 2014; Zickfeld et al., 2019), and “Kama Muta” (Fiske et al., 2019).

Self-transcendent positive emotions are generally defined as emotions that, contrary to Joy or Pride, are not bound to the immediate self-interest of those who experience them: they lead people to “transcend their own momentary needs and desires and focus on those of another” (Stellar et al., 2017, p. 201) or “direct attention firmly outside the self, away from one’s mundane expectations and immediate needs” (Shiota, Thrash, et al., 2014, p. 373). It has been proposed that some self-transcendent emotions (such as Awe and Elevation) contribute to increasing our well-being through motivating prosocial behavior (Stellar et al., 2017), while others, such as Interest and Curiosity, have been claimed to belong to a motivational system independent from pleasure that motivates us to gather new information (Hsee & Ruan, 2016).

Though the label “self-transcendent emotions” is often used as a theoretical category, it is not clear whether this constitutes a meaningful construct that captures a unified class of emotions that share core properties. Indeed, one limitation of this growing literature on positive self-transcendent emotions is that candidate emotions have mostly been studied in isolation. As an exception, Algoe & Haidt (2009) elicited and compared three self-transcendent emotions (Admiration, Gratitude, and Elevation), and observed that even if each of these emotions have characteristic features of their own, they also share a *family resemblance*—i.e., properties that distinguished them from the “happiness family” (see also Siegel et al., 2014). However, this only covers a subset of the numerous candidate self-transcendent emotions.

One way to address this problem would be to produce a taxonomy of positive emotions and see whether self-transcendent emotions constitute a group of their own, or whether other classifications are more useful to carve the space of positive emotions. Emotion taxonomies can adopt a *top-down* or a *bottom-up* approach. In a *top-down* approach, researchers identify one or more important theoretical features of emotions and divide the emotional space into different classes according to them. For example, Shiota, Neufeld, and colleagues (2014) developed the PANACEAS, a fine-grained top-down taxonomy, in which discrete positive emotions are classified depending on the kind of opportunities they respond to. By contrast, in a *bottom-up* approach, researchers group emotions together based on statistical methods, such as exploratory factor analysis, without precisely identifying the theoretical criteria and conceptual dimensions that separate these different emotions from each other. For example, using self-reported measures as empirical evidence for fine-grained distinctions, Weidman & Tracy (2020) identified nine distinct discrete positive emotions; Cowen & Keltner (2017), twenty-seven discrete emotions.

In this research, we decided to take a *bottom-up* approach and to investigate whether the label “self-transcendent emotions” captures a family of emotions in which all members share a common subjective feeling. Indeed, the expression “emotion family” has been used to divide the emotional space on the basis of what emotions feel like rather than on the basis of conceptual distinctions between emotions—the assumption being that if emotions feel the same, this is because they serve similar functions (Fredrickson, 1998). We decided to base our classification primarily on the occurrence of emotional labels, but still investigated the cognitive appraisals, bodily feelings, and action tendencies associated to each of the emotion families we identified. Even if the subjective feeling captured by the self-report measures can be considered as just the conscious reflection of all the emotional components (Grandjean et al., 2008), some methodological doubts have been raised about the possibility of leaving subjective feelings out of the construction of emotion taxonomies (Kron, 2019).

To produce this bottom-up taxonomy of self-transcendent positive emotions, based on participants’ subjective experience of positive emotions (as reflected in their use of emotional labels), we used principal component analysis on two large datasets in which participants confronted with various emotional situations reported the emotions, bodily feelings, action tendencies, and cognitive appraisals they experienced. Inducing different emotions is a technique that aims to have a more complete grasp on the emotional space and has already been used in previous bottom-up taxonomies of emotions (Cowen & Keltner, 2017; Weidman & Tracy, 2020). One dataset used an episodic recall paradigm (study 1) on a large variety of emotions, while the second used video clips known to elicit typical self-transcendent emotions (study 2), but both used similar measures. The design of study 1 allows us to get a picture of how people experience different kinds of positive emotions, and whether they make a distinction between self-transcendent and non-self-transcendent positive emotions, while study 2 focuses specifically on the elicitation of self-transcendent emotions.

In both studies, our main research question was whether the various candidate self-transcendent emotions found in the literature (Admiration, Awe, Being Moved/Kama Muta, Compassion, Elevation, Gratitude, Inspiration, Wonder) would form a unified category and share a common subjective feeling, or if it would be possible to identify different families of self-transcendent emotions based on participants’ subjective experience, as expressed in their use of positive emotion labels.

## Study 1

Our first dataset (the Geneva Positive Emotions Dataset I) was composed using a widespread method in the study of self-transcendent emotions: we instructed participants to remember an episode of their life in which they experienced a certain emotion or a certain situation supposed to elicit the target emotion.

### Method

#### Participants

A total of 3,219 English-speaking participants without any residency limitation were recruited through Prolific Academic. After exclusion based on irrelevant or meaningless open-ended answers and two attention checks, we were left with 3,113 participants (1,749 men, 1,340 women, 24 others; *M*_age_ = 26.78, *SD*_age_ = 9.24). Our goal was to recruit around 200 participants for each experimental condition involving self-transcendent emotions, and 100 for each condition involving other emotions.

#### Materials and Procedure

##### *Recall Task*

Participants were asked to remember and describe in a few lines a moment in their life when they experienced a certain emotion. As our goal was to measure emotional reactions in a wide variety of situations to determine which ones are more likely to covariate, we tried to have an exhaustive grasp on the emotional space. Target emotions were selected from a series of self-transcendent positive emotions (Admiration, Awe, Being Moved, Compassion, Elevation, or Gratitude) and non-self-transcendent emotions (Amusement, Contempt, Disgust, Fear, Joy, Pride, Sadness, Surprise). There were 17 different formulations for the recall task, based on a survey of the psychological literature (see Appendix A). As usually done in the literature on self-transcendent emotions, recall tasks were of three kinds: (i) some identified the emotional episodes by directly using the relevant emotional label, (ii) others avoided using emotional labels and rather described the kind of situation most likely to trigger the target emotion, while (iii) others used a blend of both approaches.

##### Basic Affective Dimensions

Participants were asked to rate their experience at the time on five general dimensions: Valence, Control, Arousal, Impact (see Sacharin et al., 2012) and Pleasantness (on a scale from –5 = “Very Negative/Little/Calm/Weak/Unpleasant” to 5 = “Very Positive/Much/Stimulating-Arousing/Strong/Pleasant”: see Appendix C). The Pleasantness question was introduced while data collection was under way. Because of that and because the measures of Valence and Pleasantness were highly correlated (*r* = .91), we only used Valence in our analyses.

##### *Emotional Labels*

Participants were presented with 40 emotional labels (see Appendix B). They were asked to think about the situation they just described and indicated to which extent they felt each emotion in this moment (on a scale from 0 = “Not at all” to 6 = “Very strongly”). One label (Admiration) was introduced during recruitment (*N* = 1,336) and excluded from analyses.

##### *Bodily Feelings*

Participants were presented with 18 bodily feelings and asked to which extent they experienced them in the remembered situation (on a scale from 0 = “Not at all” to 6 = “Very strongly”). A principal component analysis (PCA) with Varimax Rotation distinguished five main dimensions of bodily feelings: “Relax” (e.g., “*muscles relaxed*”), “Crying” (e.g., “*tears in my eyes*”), “Shock” (e.g., “*I felt my jaw drop*”), “Chills” (e.g., “*chills or shivers*”), and “Activation” (e.g., “*increased heart rate*”). Details can be found in Appendix D.

##### *Appraisals*

Participants were then presented with 44 items probing their cognitive state at the moment and asked to which extent they had the corresponding feelings or thoughts in the remembered situation (on a scale from 0 = “Not at all” to 6 = “Very strongly”). They were also presented with 14 statements about the situation they remembered and asked to rate their agreement with each statement (on a scale from –3 = “Fully disagree” to 3 = “Fully agree”). The items were all presented in the literature as measuring properties of one or several self-transcendent positive emotions (for details, see Appendix E). A PCA with Varimax Rotation was conducted on the 58 items and identified eight main dimensions of cognitive and situation appraisals: “Contact with Something Greater” (e.g., “I felt the presence of something greater than myself”), “Witnessing Outstanding Standard” (e.g., “The situation showed how people can go beyond themselves”), “Small Self” (e.g., “I felt that my sense of self was diminished”), “Situational Valence” (e.g., “It was unfair”), “Feeling of Social Connection” (e.g., “I felt more strongly committed to a relationship”), “Time Perception” (e.g., “I noticed time slowing”), “Mental Challenge” (e.g., “I felt challenged to mentally process what I was experiencing”), and “Optimism about Humanity” (e.g., “I felt optimistic about humanity”). Details can be found in Appendix E.

##### *Action Tendencies*

Participants were presented with 19 action tendencies and asked to which extent they experienced them in the remembered situation (on a scale from 0 = “Not at all” to 6 = “Very strongly”). The 4 last items were introduced during data collection and excluded from analyses. A PCA with Varimax Rotation identified three main dimensions: “Prosocial action tendencies” (e.g., “Telling someone how much I care about them”), “Self-Enhancing action tendencies” (e.g., “Engaging in activities that would lead to professional or academic success”), and “Enjoy” (e.g., “Laughing”). Details can be found in Appendix F.

##### *Other Measures*

We also collected other data that we do not analyse or use in the present paper and report in Appendix I.

##### *Transparency and Openness*

We report all data exclusions, all manipulations, and all measures in the study. All data, analysis code, and research materials are available at 10.17605/OSF.IO/Y9NZA.

### Results

#### Emotional Labels

To identify the main emotional dimensions, we applied a principal component analysis on emotional labels. This particular case of factor analysis fits well the conceptual and statistical ground of the data, as we had no hypothesis on the existence of a latent construct beyond the labelled emotions, the correlations between the variables are almost all significant, and the number of variables is consistent (Gorsuch, 1990, 1997). The goal of PCA is to reduce the information given by the ensemble of the items and to create components supposed to reflect what is already measured by the items. In a nutshell, it looks at the activation patterns of the 39 emotions, search for their correlations, and determine whether there is one or more latent dimensions that make emotions vary altogether.

To decide the number of components to retain, we first applied the common Kaiser’s method (eigenvalue > 1), which suggested the existence of 6 different components. Therefore, 6 dimensions, explaining 67.52% of the total variance, were retained to reduce the information of the 39 emotional labels. As our goals were exclusively exploratory, we chose an orthogonal rotation that produces uncorrelated components that are more easily interpretable as they allow to better differentiate the families of emotions.

The solution in six dimensions seems to capture six coherent emotion families: three families of positive emotions and three families of negative emotions (Table 1). Loadings above .50 were considered good measures of the dimensions and below .32 they were ignored and are not displayed in the table for greater clarity (Tabachnick & Fidell, 2013). The first general component, explaining 20.8% of the total variance of the items, seems to capture the variance related to an “Hedonic State”. The second one, explaining 11.8% of the total variance of the items, seems to capture a “Social State”, and the third dimension, explaining 11.5% of the total variance, captures an “Antagonist State”. The fourth component, explaining 10.1% of the variance, seems to capture an “Epistemic State”. The fifth, explaining 7% of the total variance, captures an “Apprehensive State”. And the last, explaining 6.1% of the total variance, seems to capture “Self-evaluative Negative State”. Pattern plots are reported in Appendix B.

**Table 1:** Rotated component matrix solution of PCA on labels of emotional states (study 1)

	Correlation with valence	Component					
		1	2	3	4	5	6
Happiness	.86*	**.764**					
Joy	.83*	**.757**			.329		
Enthusiasm	.77*	**.715**			.444		
Grateful	.70*	**.706**	.455				
Thankful	.69*	**.700**	.458				
Excitement	.72*	**.690**			.471		
Well-Being	.73*	**.675**					
Contentment	.65*	**.665**					
Uplifted	.69*	**.635**	.325				
Appreciative	.68*	**.625**	.482				
Pride	.55*	**.598**					
Sadness	− .72*	** − .578**		.425			
Amusement	.54*	**.549**			.490		
Inspiration	.66*	**.511**	.449		.364		
Touched	.36*		**.742**				
Compassion	.11*		**.730**				
Moved	.37*		**.715**				
Tenderness	.34*		**.660**				
Love	.48*	.434	**.610**				
Respect	.52*	.348	**.608**				
Outrage	− .47*			**.759**			
Indignation	− .54*			**.729**			
Hate	− .62*			**.715**			
Disgust	− .64*			**.685**			
Anger	− .73*	− .444		**.659**			
Injustice	− .63*	− .459		**.644**			
Contempt	− .05			**.613**			
Curious	.38*				**.781**		
Wonder	.51*	.322			**.638**		
Interest	.56*	.366	.324		**.621**		
Fascination	.64*	.473			**.619**		
Surprise	.30*				**.528**		
Awe	.23*				.492		
Nervous	− .40*					**.832**	
Anxiety	− .50*					**.831**	
Fear	− .54*					**.792**	
Embarrassed	− .32*						**.805**
Shame	− .44*			.320			**.784**
Guilt	− .38*						**.686**

Left column indicates the correlation between each emotional label and valence. *p < .001. Bold: loadings > .50

Awe was the only emotion that did not reach the loading of .50 in any components. Indeed, when looking at extraction communalities (see Appendix H), Awe, Surprise, and Contempt variances seem to be poorly explained by the variance of the extracted dimensions, *h*^2^ < .50. Eventually, extraction communality of Awe would considerably increase (> .80) when extracting seven dimensions instead of six, but the seventh dimension would be weak and unstable because Awe is the only emotional label loading above .40 on it (Costello & Osborne, 2005).

The first family, “Hedonic states”, shows the strongest relationship to our measure of valence and seems characterized by emotions that are pleasant (Amusement) or directly tied to one’s self-interests and goals (Happiness, Contentment, Pride). Most self-transcendent emotions loaded either on the Social or the Epistemic states dimensions.

#### Relationships Between Emotional Dimensions and Other Measures

An important advantage of using principal component analysis is the possibility of creating factor scores. These are estimations of the position of every participant on the dimensions extracted by the analysis. It allows us to reduce the amount of detailed information given by the separate emotional labels and to focus on the states related to the different emotion families. Factor scores were created using the regression approach and they have a mean of 0 and a standard deviation of 1. As they are estimations of orthogonal dimensions, the correlations between factor scores are equal to 0.

**Fig. 1:**
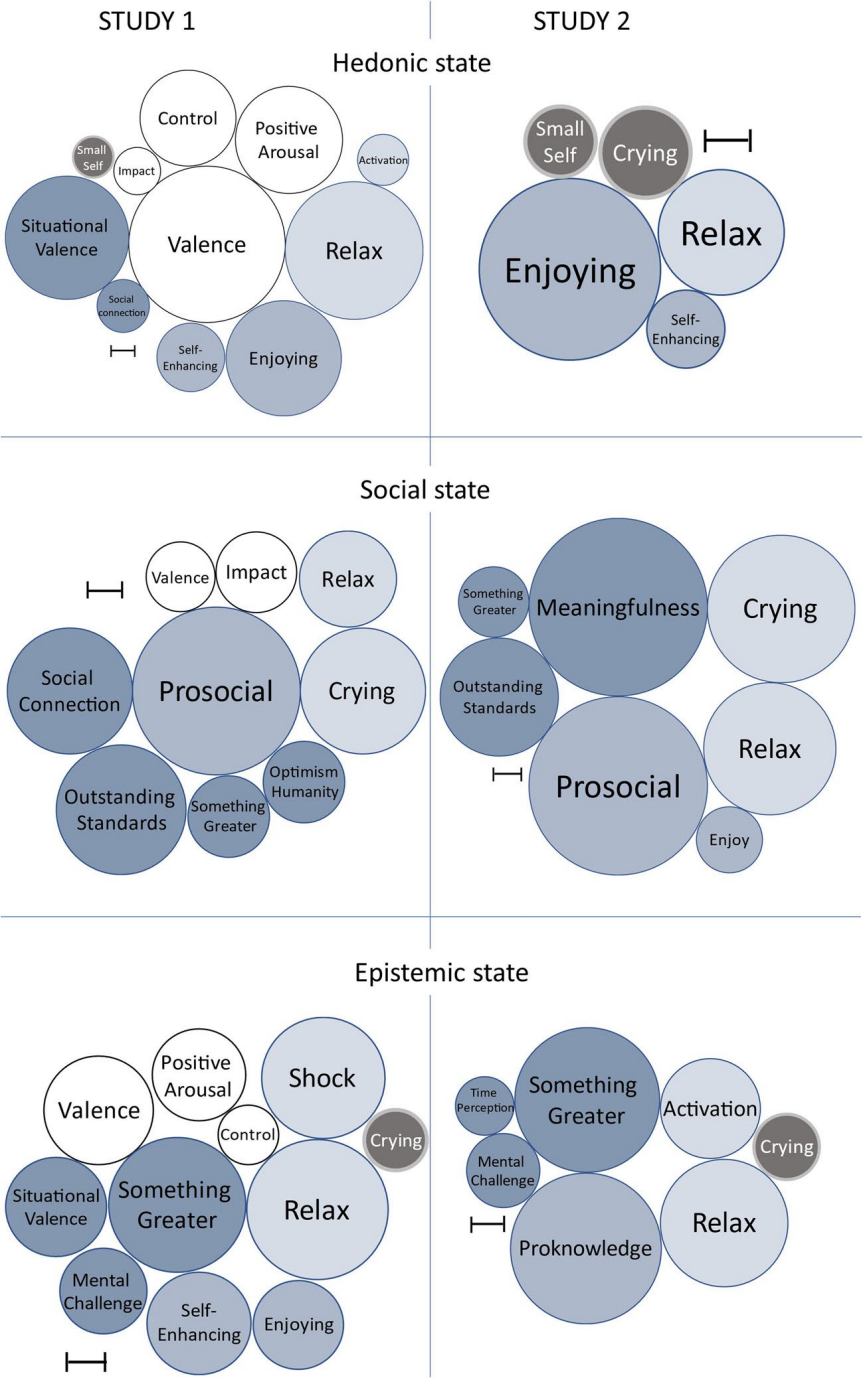
Graphic representation of correlations between Emotion Families and Other Emotional Components. In this figure, we only show the strongest correlations (*r* ≥  ± .15) reported in Table 2 and Table 4. Light-colored circles signal a positive correlation; dark-coloured circles signal a negative correlation. The dimension of the circles is proportionate to the strength of the correlation; the arrow represents the measure corresponding to a *r* = .10 in each different representation

To assess the general features of the 6 dimensions, we looked at their correlations with the other measures we collected: basic affective dimensions, bodily feelings, action tendencies, and appraisals. Results are presented in Table 2. As can be seen, each of the three families of positive emotions presents distinguishing characteristics. These characteristics are summarized in Fig. 1.

**Table 2:** Correlations between six emotion families and other emotional components (study 1)

	Hedonic	Social	Epistemic	Antagonist	Apprehensive	Negative self-evaluation
*Basic affective dimensions*						
Valence	.664* [.638,.690]	.202* [.158,.246]	.273* [.230,.316]	− .376* [− .416, − .336]	− .274* [− .317, − .231]	− .146* [− .191, − .101]
Control	.408* [.370, .447]	.036 [− .010, .082]	.150* [.105, .195]	− .132* [− .177, − .087]	− .213* [− .257, − .169]	− .176* [− .221, − .131]
Positive Arousal	.455* [.418, .491]	.055* [.009, .101]	.233* [.189, .277]	− .144* [− .189, − .099]	− .068* [− .114, − .022]	− .092* [− .138, − .046]
Impact	.207* [.163, .251]	.235* [.191, .279]	.087* [.041, .133]	.010 [− .036, .056]	.125* [.080, .171]	− .015 [− .061, .031]
*Bodily feelings*						
Relax	.584* [.554, .614]	.282* [.240, .325]	.307* [.265, .349]	− .170* [− .215, − .125]	− .224* [− .268, − .180]	− .103* [− .149, − .057]
Crying	− .081* [− .127, − .035]	.367* [.327, .407]	− .155* [− .200, − .110]	.165* [.120, .210]	.197* [.153, .241]	.133* [.088, .178]
Shock	.058* [.012, .100]	− .001 [− .047, .045]	.353* [.313, .393]	.138* [.093, .183]	.070* [.024, .116]	.001 [− .045, .047]
Chills	− .113* [− .159, − .067]	− .006 [− .052, .040]	.146* [.101, .191]	.194* [.150, .239]	.308* [.266, .350]	.030 [− .016, .076]
Activation	.218* [.174, .262]	− .065* [− .111, − .019]	.043 [− .003, .089]	.011 [− .035, .057]	.352* [.311, .393]	.151* [.106, .196]
*Action tendencies*						
Prosocial	.052* [.010, .098]	.545* [.513, .578]	− .059* [− .105, − .013]	.066* [.020, .112]	.068* [.022, .114]	.058* [.012, .104]
Self-Enhancing	.290* [.248, .332]	.111* [.065, .157]	.260* [.217, .303]	.067* [.021, .113]	.019 [− .027, .065]	.047* [.001, .093]
Enjoy	.489* [.454, .524]	.039 [− .007, .085]	.225* [.181, .269]	− .178* [− .222, − .133]	− .061* [− .107, − .015]	− .128* [− .173, − .083]
*Appraisals*						
Within Something Greater	.143* [.108, .177]	.239* [.206, .272]	.340* [.309, .371]	− .001 [− .047, .045]	− .004 [− .05, .042]	− .172* [− .217, − .127]
Outstanding Standards	.144* [.109, .178]	.379* [.349, .409]	.006 [− .029, .041]	− .062* [− .108, − .016]	.022 [− .024, .068]	.116* [.07, .161]
Small Self	− .173* [− .207, − .139]	.097* [.062, .132]	.054* [.019, .089]	.225* [.181, .269]	.133* [.087, .178]	.236* [.192, .28]
Situational Valence	.524* [.498, .549]	.069* [.034, .104]	.251* [.218, .284]	− .407* [− .446, − .369]	− .280* [− .323, − .237]	− .043 [− .089, .003]
Social Connection	.224* [.19, .257]	.366* [.335, .396]	− .054* [− .089, − .019]	.092* [.046, .138]	.033 [− .013, .079]	− .046 [− .092, .0001]
Time Perception	.092* [.057, .127]	− .048* [− .083, − .013]	.100* [.065, .135]	.025 [− .021, .071]	.210* [.166, .254]	.017 [− .029, .063]
Mental Challenge	.032 [− .003, .067]	.036 [.001, .071]	.217* [.183, .25]	.116* [.070, .162]	.235* [.191, .279]	.099* [.053, .145]
Optimism about humanity	.115* [.08, .15]	.187* [.153, .221]	− .102* [− .137, − .067]	.014 [− .032, .060]	− .046* [− .092, − .0001]	.083* [.037, .129]

Brackets contain 99% confidence intervals of correlations

^*^ *p* < .001

### Discussion

Our results suggest that three different positive emotion families can be differentiated: Hedonic states, Social states, and Epistemic states. Given that most candidate self-transcendent emotions loaded primarily either on the Social and Epistemic dimensions, while typical non-self-transcendent (i.e., self-directed) emotions loaded primarily on the Hedonic dimension, this suggests (i) that self-transcendent emotions can be empirically distinguished from non-self-transcendent emotions, and (ii) that self-transcendent emotions can be divided into at least two main families characterized by different subjective feelings and properties.

## Study 2

Our second dataset (the Geneva Positive Emotions Dataset II) was composed using another widespread method in the study of self-transcendent emotions: we presented participants with short video clips supposed to elicit the target emotion.

### Method

#### Participants

Participants were 1,578 US residents recruited through Prolific Academic. After exclusion based on irrelevant or meaningless open-ended answers and two attention checks, we were left with 1,443 participants (727 men, 697 women, 19 others; *M*_age_ = 37.43, *SD*_age_ = 13.67). Our goal was to recruit around 200 participants for each target emotion.

#### Materials and Procedure

##### *Video Clips*

The study took the form of an online survey. Participants were first asked to watch a short video clip. There were six target emotions (Neutral, Amusement, Nature-elicited Awe, Other-elicited Awe, Altruism-elicited Being Moved, and Other-elicited Being Moved) and three different videos for each of them, for a total of 18 videos. Video selection was based on a survey of the psychological literature on self-transcendent emotions, and 16 out of 18 were used in previous studies to induce specific emotions (see Appendix A).

Just after watching the video clip, participants were told that a certain number of them would receive a £50 bonus and were asked to decide now how much of this bonus they were willing to give to four charities (selected in a list of 12 charities). The goal of this question was to assess the impact of emotion induction on charitable behavior and the results for this measure are described in Cova (2023).

Then, participants were asked (i) to describe what the video was about, (ii) the part they remember more vividly, and (iii) to provide five separate words describing their emotional state while watching the video.

##### *Emotional Labels*

Participants were presented with the same 40 emotional labels used in study 1 (see Appendix B) and had to indicate to which extent they felt each emotion while watching the video (on a scale from 0 = “Not at all” to 6 = “Very strongly”).

##### *Bodily Feelings*

Participants were presented with the same bodily feelings as in study 1 and asked to indicate to which extent they experienced them while watching the part of the video they remembered more vividly (on a scale from 0 = “Not at all” to 6 = “Very strongly”). A principal component analysis (PCA) with Varimax Rotation distinguished three main dimensions of bodily feelings: “Activation” (e.g., “increased heart rate”, “I felt my jaw drop”), “Crying” (e.g., “tears in my eyes”), and “Relax” (e.g., “muscles relaxed”). Details can be found in Appendix D.

##### *Appraisals*

Participants were presented with 48 items about their cognitive state and asked to indicate to which extent they had the corresponding feelings or thoughts while watching the part of the video they remembered more vividly (on a scale from 0 = “Not at all” to 6 = “Very strongly”). All measures included in study 1 were used also in this study, except for six items that did not load on any dimension in study 1. Also, we added eight items about the perceived meaningfulness of the situation and two items about self-improvement. As in study 1, we presented participants with statements about the situation presented on the video and asked to them to rate their agreement (on a scale from -3 = “Fully disagree” to 3 = “Fully agree”). Six statements present in study 1 were included in this study. A PCA with Varimax Rotation was conducted on the 54 items and identified six main dimensions of cognitive and situation appraisals: “Meaningfulness” (e.g., “I realized that I should positively contribute to something beyond myself”), “Contact with Something Greater” (e.g., “I felt the existence of things more powerful than myself”), “Witnessing Outstanding Standard” (e.g., “The situation showed how people can go beyond themselves”), “Small Self” (e.g., “I felt that my sense of self was diminished”), “Time Perception” (e.g., “I noticed time slowing”), and “Mental Challenge” (e.g., “I felt challenged to mentally process what I was experiencing”). Details can be found in Appendix E.

##### *Action Tendencies*

Participants were presented with 27 action tendencies and asked to which extent they experienced them while watching the video or just after (on a scale from 0 = “Not at all” to 6 = “Very strongly”). All measures used in study 1 were included, except for one item (“Emulating”) that did not load on any specific dimension. We also added three items related to learning and creating and several items measuring participants’ intentions to help people in need, environment, artists, or scientists. A PCA with Varimax Rotation was conducted on the 27 items and it identified four main dimensions: “Prosocial action tendencies” (e.g., “Telling someone how much I care about them”), “Proknowledge action tendencies” (e.g., “Supporting scientists”), “Self-Enhancing action tendencies” (e.g., “Engaging in activities that would lead to professional or academic success”), and “Enjoy” (e.g., “Laughing”). Details can be found in Appendix F.

##### *Other Measures*

We also collected other data that we do not analyse or use in the present paper and report in Appendix I.

##### *Transparency and Openness*

We report all data exclusions, all manipulations, and all measures in the study. All data, analysis code, and research materials are available at 10.17605/OSF.IO/Y9NZA.

### Results

#### Emotional Labels

As in study 1, we applied a PCA with orthogonal rotation on emotional labels. We forced the analysis to extract six dimensions and when controlling, the common Kaiser’s method (eigenvalue > 1) also suggested the existence of 6 different components. Therefore, 6 dimensions, explaining 67.52% of the total variance, were retained to reduce the information of the 40 emotional labels.

Again, the solution in six dimensions seems to capture six coherent emotion families: three families of positive emotions and three families of negative emotions (Table 3). The first general component, explaining 35.6% of the total variance of the items, captures the variance related to a “Social State”. The second one, explaining 15% of the total variance of the items, captures an “Epistemic State”, and the third dimension, explaining 7.2% of the total variance, captures an “Antagonist State”. The fourth principal component, explaining 4.9% of the variance, captures an “Apprehensive State”. The fifth, explaining 2.7% of the total variance, captures an “Hedonic State”. And the last, explaining 2.6% of the total variance, captures a “Self-evaluative Negative State”. Pattern plots are reported in Appendix B. When looking at extraction communalities (see Appendix H), Surprise seems to be poorly explained by the variance of the extracted dimensions, *h*^2^ < .50.

**Table 3:** Rotated component matrix solution of PCA on labels of emotional states (study 2)

	Component					
	1	2	3	4	5	6
Touched	**.898**					
Tenderness	**.855**					
Love	**.852**					
Thankful	**.832**					
Moved	**.829**					
Compassion	**.827**					
Grateful	**.825**					
Uplifted	**.805**					
Appreciative	**.784**	.357				
Inspiration	**.750**	.423				
Respect	**.726**					
Admiration	**.714**	.423				
Joy	**.705**				.362	
Well-Being	**.696**					
Happiness	**.691**				.364	
Pride	**.654**					
Contentment	.479	.321			.324	
Fascination		**.814**				
Curious		**.764**				
Wonder	.372	**.764**				
Interest	.394	**.690**				
Awe	.489	**.663**				
Excitement	.402	**.595**			.417	
Enthusiasm	**.528**	**.532**			.363	
Surprise		.474				
Anger			**.809**			
Outrage			**.770**			
Disgust			**.703**			
Hate			**.686**			
Indignation			**.656**			
Injustice			**.624**			
Contempt			**.505**		.474	
Nervous				**.845**		
Anxiety				**.828**		
Fear				**.801**		
Sadness	.352		.348	.464	− .342	
Amusement					**.683**	
Shame			.381			**.765**
Embarrassed						**.730**
Guilt			.328			**.715**

Bold: loadings > .50

#### Relationships Between Emotional Dimensions and Other Measures

We followed the same procedure as in study 1 and we created factor scores based on the dimensions extracted by the PCA and looked at their correlations with the other measures we collected. Results are presented in Table 4. As can be seen in Fig. 1, the characteristics that distinguish each family of positive emotions from the others were compatible with the results of study 1.

**Table 4:** Correlations between six emotion families and other emotional components (study 2)

	Hedonic	Social	Epistemic	Antagonist	Apprehensive	Negative self-evaluation
*Bodily feelings*						
Excitation	.055 [− .013; .123]	.043 [− .025, .111]	.308* [.247, .369]	.270* [.207, .333]	.489* [.437, .541]	.135* [.068, .202]
Crying	− .200* [− .265, − .135]	.532* [.483,.581]	− .201* [− .266, − .136]	.088* [.021,.155]	.097* [.03,.164]	.072* [.05,.14]
Relax	.278* [.215,.341]	.478* [.426,.53]	.390* [.332,.448]	− .111* [− .178, − .044]	− .249* [− .313, − .185]	− .047 [− .115,.021]
*Action tendencies*						
Prosocial	− .124* [− .191, − .057]	.641* [.601,.681]	− .088* [− .155, − .021]	.082* [.015,.15]	.162* [.096,.228]	.032 [− .036,.1]
Proknowledge	.038 [− .03,.101]	.127* [.06,.194]	.463* [.401,.516]	− .001 [− .069,.067]	.142* [.076,.201]	− .002 [− .07,.066]
Self-Enhancing	.172* [.106,.238]	.108* [.041,.175]	.131* [.064,.198]	.077* [.01,.145]	.007 [− .061,.075]	.026 [− .042,.094]
Enjoy	.402* [.345,.459]	.238* [.174,.302]	.075* [.008,.143]	− .031 [− .099,.037]	− .113* [− .18, − .046]	− .007 [− .075,.061]
*Cognitive and situation appraisals*						
Meaningfulness	.088* [.021,.155]	.641* [.601,.681]	.045 [− .023,.113]	.095* [.028,.162]	.016 [− .052,.084]	.044 [− .024,.112]
Within Something Greater	− .082* [− .149, − .015]	.254* [.191,.318]	.445* [.391,.499]	− .121* [− .188, − .054]	.050 [− .018,.118]	− .129* [− .196, − .062]
Outstanding Standard	.010 [− .058,.078]	.426* [.37,.462]	− .100* [− .167, − .033]	− .017 [− .085,.051]	− .010 [− .078,.058]	− .023 [− .091,.045]
Small Self	− .156* [− .222, − .09]	.041 [− .027,.109]	.090* [.023,.157]	.133* [.066,.12]	.327* [.266,.388]	.222* [.158,.287]
Time Perception	.101* [.034,.168]	.047 [− .021,.115]	.180* [.114,.246]	− .029 [− .097, − .039]	.020 [− .048,.088]	− .021 [− .089,.047]
Mental Challenge	.037 [− .031,.105]	− .013 [− .081,.055]	.227* [.163,.291]	.102* [.035,.169]	.266* [.203,.329]	.038 [− .03,.106]

Brackets contain 99% confidence intervals of correlations

^*^p < .001

### Discussion

Results from study 2 suggest that the main findings of study 1 are stable: the emotional space was again divided into the same six dimensions of affective states, including three positive dimensions; and most positive self-transcendent emotions loaded onto two dimensions: Social and Epistemic.

One key difference between the two studies, though, was that the “Hedonic” dimension was considerably less represented than in study 1. We think this was because the “Hedonic” from study 1 mostly captured a positive state connected to participants’ own well-being and self-interests (i.e., non-self-transcendent emotions). However, in study 2, participants were asked to react to events happening to other (sometimes fictional) people. It is thus not surprising that emotional reactions directly connected to their self-interest were rare (with the exception of Amusement), and that most positive emotional labels fell into the Social category: most positive emotions (such as Joy or Pride) were felt *for others* and thus loaded on the other-directed Social dimension. Thus, despite some differences with study 1, the results of study 2 seem to confirm the distinction between Social and Epistemic self-transcendent emotions.

## General Discussion

In this paper, we investigated how typical candidate positive self-transcendent emotions were scattered across the space of positive and negative emotions based on their subjective feelings.

We identified three different positive emotions dimensions. The first dimension (*Hedonic state*) was positively associated with bodily feelings of Activation and Relaxation and the motivation to Enjoy oneself and to engage in Self-Enhancing behaviors. Most paradigmatic non-self-transcendent positive emotions, such as Happiness, Joy, or Pride loaded on this dimension, suggesting that it captured self-centred emotions. Typical self-transcendent emotions loaded primarily on the two other positive dimensions. The second dimension (*Social state*) was associated with more tearful bodily feelings (while still being positively valenced), and connected to concerns about Social Connection, Outstanding Standards, and Meaningfulness and the motivation to engage in prosocial behavior. It captured emotions such as “Being moved”, “Being touched”, “Love”, and “Compassion”. The third dimension (*Epistemic state*) was associated with Shock, Relaxation, and Mental Challenge, while still being pleasant. It was also associated with the feeling of being in the presence of Something Greater and with Slowed Time Perception, as well as motivation to engage with and learn new things. It captured emotions such as Awe, Interest, Surprise, and Wonder.

We decided to use for each family of emotions a name that refers to the kind of values around which the appraisals characteristic of these emotions revolve. In the Hedonic family of emotions, the prevalent feature is the hedonic value of the experience, in the sense that what brings together those emotions is that the value of self-centred pleasantness is important in the situation (Leach, 2019). The Social family of emotions brings together emotions that are elicited in situations in which social values are at stake (Algoe & Haidt, 2009; Haidt, 2003b; Landmann et al., 2019; Seibt et al., 2017; Thomson & Siegel, 2017). Finally, emotions in the Epistemic family resemble one another in that they are elicited in situations in which epistemic values are important (Anderson et al., 2020; Gottlieb et al., 2018; Keltner & Shiota, 2003; McPhetres, 2019; Pekrun et al., 2017; Shiota et al., 2007, Shiota, Neufeld, et al., 2014; Shiota, Thrash, et al., 2014; Silvia, 2008).

As such, our results suggest that self-transcendent and non-self-transcendent emotions can be distinguished on an empirical basis, as both types of emotions tended to load on separate dimensions. Moreover, our results suggest that self-transcendent emotions can be divided into at least two broad families that serve two distinct functions: Social emotions, which motivate us to engage in prosocial behavior; and Epistemic emotions, which motivate us to seek and engage with new information. Both families of self-transcendent emotions are characterized by different bodily feelings, cognitive appraisals, and action tendencies.

Our research’s major limitation is the lack of physiological measures and behavioral tasks to assess emotional components, which prevents us from drawing conclusions about the actual physiological and behavioral changes linked to each emotion family. Because the research focuses on self-reported measures and English-speaking samples, our results do not consider possible cross-cultural differences (some cultures could for instance give less importance than others to abstract knowledge) that could be explored in future research. Moreover, our list of items is limited to bodily feelings, cognitive appraisals, and action tendencies that have typically been associated with self-transcendent emotions. As such, it is not exhaustive, and we might have missed important features of our emotion families.

We used labels to identify emotions, which means that, depending on the context, some labels could be more or less related to one emotion family or another. Moreover, different sets of emotional labels may lead to a different partition of the emotional space: in this taxonomy, the focus was the contrast in feelings between self-centred and self-transcendent emotions, and between different self-transcendent emotions. The interpretation of our results should be limited to that (Desmet et al., 2021). Nevertheless, these results seem robust; when analyses are limited to self-transcendent emotional labels, the emotional space is divided into the same emotional dimensions (see Appendix J).

Finally, it should be acknowledged that our taxonomy of positive (and self-transcendent) emotions is *coarse-grained*. While some authors are interested in fine-grained differentiations of discrete positive emotions (Cowen & Keltner, 2017; Desmet, 2012; Shiota et al., 2017; Weidman & Tracy, 2020; Yih et al., 2020), others are interested in coarse-grained differentiation of clusters, or families, of positive emotions (Algoe & Haidt, 2009; Fredrickson, 1998; Sauter, 2017; Shiota, Neufeld, et al., 2014; Shiota, Thrash, et al., 2014), and our paper took the latter approach. As such, our results do not allow to distinguish between particular self-transcendent emotions within each family. Still, we think that our results provide a first step in an empirical, *bottom-up* classification of self-transcendent emotion even though further research will be needed to determine how emotions within each family should be distinguished from each other.

### Supplementary Information

Below is the link to the electronic supplementary material.

Supplementary file1 (DOCX 334 KB)

## Additional Information

### Data availability

The datasets generated during and/or analysed during both studies are available in the Open Science Framework (OSF) repository, 10.17605/OSF.IO/Y9NZA.
