# Supplementary material for: Are Self-transcendent Emotions One Big Family? An Empirical Taxonomy of Positive Self-transcendent Emotion Labels
Source: Affect Sci. 2023 Jun 19;4(4):731–43. doi: 10.1007/s42761-023-00194-1 (PMC10751273; doi:10.1007/s42761-023-00194-1)
Supplement: Supplementary file 1 — Supplementary file1 (DOCX 334 KB) [file 42761_2023_194_MOESM1_ESM.docx]

Appendices - Are self-transcendent emotions one big family? An empirical taxonomy of positive self-transcendent emotion labels

Angela Gaia F. Abatista^abc^* and Florian Cova^ab^

^a^Swiss Center for Affective Sciences, University of Geneva, Switzerland; ^b^Department of Philosophy, University of Geneva, Switzerland; ^c^ Psychology Department, Université Grenoble Alpes, France

*Angela Gaia F. Abatista angela.abatista@unige.ch Swiss Center for Affective Sciences, University of Geneva, Campus Biotech, Chemin des Mines 9, CH-1202 Genève

https://orcid.org/0000-0003-4503-5197

Florian Cova [florian.cova@gmail.com](mailto:florian.cova@gmail.com) Department of Philosophy, University of Geneva, rue de Candolle 2, CH-1205 Genève 4

https://orcid.org/0000-0001-9360-8290

# Appendix A

## Study 1

Instructions could be either *Labelled* (= simply giving participants the name of the target emotion; e.g. “remember a particular time in your life when you felt awe.”) or *Description* (= giving participants a more detailed specification of the type of situations they were asked to remember, and which might not necessarily include the name of the target emotion; e.g. “remember a particular time in your life when you saw someone demonstrating humanity’s higher or better nature”), or *Mix* (= a blend of the two approaches; e.g. “think about a particular time, fairly recently, when you encountered a stunning natural scene that caused you to feel awe”). In all conditions, participants were instructed: “Please, try to choose an event dating back from at least one year. Also, take care not to provide any information that would betray your identity or another person’s identity.” All instructions are presented in Table S1. Here is an example of full instructions for the *Gratitude – Description* condition:

Please, take a few minutes to remember a particular time in your life of a specific time **when someone did something really good for you**. Please pick a situation in which you benefited because of someone else’s kindness, helpfulness, or generosity. Then describe in a few lines the situation you just remembered.
(Please, try to choose an event dating back from at least one year. Also, take care not to provide any information that would betray your identity or another person’s identity.)

**Table S1**

*Instructions and number of participants for each condition.*

| *Emotion* | *Instruction* | *Text* |
| --- | --- | --- |
| 1. ADMIRATION | *Labelled*  N = 211 | Please, take a few minutes to remember a particular time in your life **when you felt admiration for someone**. Then describe in a few lines the situation you just remembered. |
| 1. AMUSEMENT | *Labelled*  N = 211 | Please, take a few minutes to remember a particular time in your life when you felt **amused**. It can be a situation in which you found something funny or hilarious. Then describe in a few lines the situation you just remembered. |
| 1. AWE | *Labelled*  N = 353 | Please, take a few minutes to remember a particular time in your life when you felt **awe**. Then describe in a few lines the situation you just remembered. |
|  | *Mix*  N = 313 | Please take a few minutes to think about a particular time, fairly recently, when you encountered a stunning natural scene that caused you to feel **awe**. This might have been a sunset, a view from a high place, or any other time you were in a natural setting that you felt was beautiful. Then describe in a few lines the situation you just remembered. (from Piff et al., 2015) |
| 1. BEING MOVED | *Labelled*  N = 202 | Sometimes, we say of certain things that they are **moving** or **touching**. Please, take a few minutes to remember a particular time in your life when you felt **moved** or **touched**. Then describe in a few lines the situation you just remembered. |
|  | *Description*  *(Kama Muta)*  N = 210 | Please, take a few minutes to remember a particular time in your life **when you got moist eyes and even shed a tear because of a positive feeling**. Then describe in a few lines the situation you just remembered. (from Zickfeld, Schubert, Seibt, Blomster, et al., 2019) |
| 1. COMPASSION | *Labelled*  N = 207 | Please, take a few minutes to remember a particular time in your life when you felt **compassion**. Then describe in a few lines the situation you just remembered. |
| 1. CONTEMPT | *Labelled*  N = 93 | Please, take a few minutes to remember a particular time in your life when you felt **contempt**. Then describe in a few lines the situation you just remembered. |
| 1. DISGUST | *Labelled*  N = 106 | Please, take a few minutes to remember a particular time in your life when you felt **disgust**. Then describe in a few lines the situation you just remembered. |
| 1. ELEVATION | *Description*  N = 263 | Please, take a few minutes to remember a particular time in your life when **you saw someone demonstrating humanity’s higher or better nature**. Please pick a situation in which you were not the beneficiary, that is, **you saw someone doing something good, honourable, or charitable for someone else**. Then describe in a few lines the situation you just remembered. (from Algoe & Haidt, 2009) |
| 1. FEAR | *Labelled*  N = 107 | Please, take a few minutes to remember a particular time in your life when you felt **fear**. Then describe in a few lines the situation you just remembered. |
| 1. GRATITUDE | *Labelled*  N = 217 | Please, take a few minutes to remember a particular time in your life when you felt **gratitude**. Then describe in a few lines the situation you just remembered. |
|  | *Description*  N = 206 | Please, take a few minutes to remember a particular time in your life of a specific time **when someone did something really good for you**. Please pick a situation in which you benefited because of someone else’s kindness, helpfulness, or generosity. Then describe in a few lines the situation you just remembered.(from Algoe & Haidt, 2009) |
| 1. JOY | *Labelled*  N = 104 | Please, take a few minutes to remember a particular time in your life when you felt **joy**. Then describe in a few lines the situation you just remembered. |
| 1. PRIDE | *Description*  N = 100 | Please, take a few minutes to remember a particular time in your life when you felt **pride**. This might have been being accepted to a university, winning an event or competition, or any other time that you achieved a personal accomplishment. Then describe in a few lines the situation you just remembered. (from Piff et al., 2015) |
| 1. SADNESS | *Labelled*  N = 108 | Please, take a few minutes to remember a particular time in your life when you felt **sad**. Then describe in a few lines the situation you just remembered. |
| 1. SURPRISE | *Labelled*  N = 102 | Please, take a few minutes to remember a particular time in your life when you felt **surprised**. Then describe in a few lines the situation you just remembered. |

## Study 2

The Nature-elicited Awe and Other-elicited Awe conditions used videos shown to induce feelings of Awe but videos in the Nature-elicited Awe condition focused on natural landscapes and elements, while videos in the Other-elicited Awe condition did not. Similarly, two different conditions were used to elicit feelings of being moved. The Altruism-elicited Being Moved (Elevation) and the Other-elicited Being Moved conditions used videos shown to induce feelings of being moved or touched but differed to the extent that videos in the Altruism-elicited Being Moved (Elevation) condition focused on people displaying virtuous and altruistic behaviour, while videos in the Other-elicited Being Moved condition did not. (In line with Cova et al., 2017, we treated Elevation as a special case of being moved: being moved by altruistic and moral actions.) All conditions are presented in Table S2.

**Table S2**

*Instructions and number of participants for each condition.*

| **Emotion** | **Condition** | **Eliciting Video** | **Source** |
| --- | --- | --- | --- |
| *Amusement* (N=228) | *Amusement_1* (N=79) | A live action reenactment of Road Runner. | (217s,from Cova et al., 2017) |
|  | *Amusement_2* (N=73) | A montage of nature clips from the BBC’s comedic series Walk on the Wild Side, composed of animals in their natural habitats acting and speaking in ways that are funny. | (228s, from Piff et al., 2015) |
|  | *Amusement_3* (N=76) | A 5-minutes excerpt of the British comedy Faulty Towers. | (309s, from Schnall et al., 2010) |
| *Awe – Natural* (N=247) | *Awe – Natural_1* (N=79) | A montage of nature clips from the BBC’s Planet Earth series composed of grand, sweeping shots of scenic vistas, mountains, plains, forests, and canyons. | (244s, from Piff et al., 2015) |
|  | *Awe – Natural_2* (N=85) | A montage of threatening natural phenomena, such as tornadoes and volcanoes | (300s, from Piff et al., 2015) |
|  | *Awe – Natural_3* (N=83) | A video showing panoramic views of natural landscapes including waterfalls, deserts, oceans, large rivers, and high mountains. | (173s, from Saroglou et al., 2008) |
| *Awe – Other* (N=236) | *Awe – Non-Natural_1* (N=82) | A video depicting a young heterosexual couple that was filmed at many points during the  woman’s pregnancy. The video showed the image of the fetus on a sonogram and the birth of the baby in the maternity hospital, followed by the mother holding her infant in the first minutes after childbirth. | (195s, from Saroglou et al., 2008) |
|  | *Awe – Non-Natural_2* (N=81) | A 3-minutes video taken in a laboratory setting of droplets of colored water colliding with a bowl of milk. This footage was shot at 5,000 frames per second, 200 times slower than real-time (from the Web-based series The Slow Mo Guys)—minute and intricate patterns in liquid are shown that are invisible to the naked eye under normal circumstances. | (298s, from Piff et al., 2015) |
|  | *Awe – Non-Natural_3* (N=73) | A video clip that shows the size of different astronomical objects by progressively zooming out, starting from the Earth to finally reach the size of the known universe. The clip is accompanied by music from the movie Interstellar. | (140s) |
| *Being Moved - Other* (N=250) | *Being Moved_1* (N=84) | A video showing a heterosexual couple getting old together while kissing and holding each other in their arms. | (82s, from (Zickfeld et al., 2019) |
|  | *Being Moved_2* (N=81) | A video showing a handicapped person auditioning for a casting show (X-Factor). During his audition, he tells the jury how he was adopted despite being handicapped, and ultimately wins the casting show | (346s, from Landmann et al., 2019) |
|  | *Being Moved_3* (N=85) | An excerpt from the Pixar movie Up in which an old man goes through an old photo album and thinks about the time he spent with his now deceased wife. The excerpt ends when the old man discovers a hidden message from his wife, telling him to have new adventures. | (200s, from Cova et al., 2017 |
| *Being Moved - Altruism (Elevation)* (N=256) | *Elevation_1* (N=87) | A clip from an episode of The Oprah Winfrey Show in which a musician pays tribute to his mentor and former music teacher, who had saved him from a life of gang activity and violence. | (236s, from Silvers & Haidt, 2008) |
|  | *Elevation_2* (N=83) | A video showing a man helping a wide variety of people, animals and even plants. For example, he helps a woman pushing a heavy trolley cross the road, or gives money to a poor girl, until she’s eventually able to return to school. | (175s, from Zickfeld et al., 2019) |
|  | *Elevation_3* (N=86) | A video depicting a little boy being bailed out by a cook for stealing medicine for his sick mother. Years later, the boy, now being a doctor, repays the favour on a much larger scale by paying for the cook’s medical expenses after he had a heart attack. | (182s, from Zickfeld et al., 2019) |
| *Neutral* (N=226) | *Neutral_1* (N=76) | A video of two people playing chess. | (180s, from Humbert-Droz et al., 2020) |
|  | *Neutral_2* (N=77) | A video of a man explaining how to build a kitchen countertop. | (234s, from Piff et al., 2015) |
|  | *Neutral_3* (N=73) | A video of a woman describing the mushrooms that can be found in a forest. | (215s) |

# Appendix B

**Emotion Labels**

## Study 1

Instructions: “Think about the situation you just described. In this very moment, to which extent did you feel...” [Scale from 0 (not at all) to 6 (very strongly)]

List of emotion labels for Study 1 and Study 2.

| 1. Amusement | 1. Anger |
| --- | --- |
| 1. Anxiety | 1. Appreciative |
| 1. Awe | 1. Compassion |
| 1. Contentment | 1. Contempt |
| 1. Disgust | 1. Embarrassed |
| 1. Enthusiasm | 1. Excitement |
| 1. Fascination | 1. Fear |
| 1. Feelings of injustice | 1. Grateful |
| 1. Guilt | 1. Happiness |
| 1. Hate | 1. Indignation |
| 1. Inspiration | 1. Interest |
| 1. Joy | 1. Love |
| 1. Moral outrage | 1. Moved |
| 1. Nervous | 1. Pride |
| 1. Respect | 1. Sadness |
| 1. Shame | 1. Surprise |
| 1. Tenderness | 1. Thankful |
| 1. Touched | 1. Uplifted |
| 1. Well-being | 1. Wonder |
| 1. Curious | 1. Admiration |

Because in Study 1 the measure of Admiration was introduced in the middle of the recruitment (N=1336), we decided to exclude this measure from our analyses in Study 1.

Results of Principal Component Analysis on 39 emotional labels representing the emotional
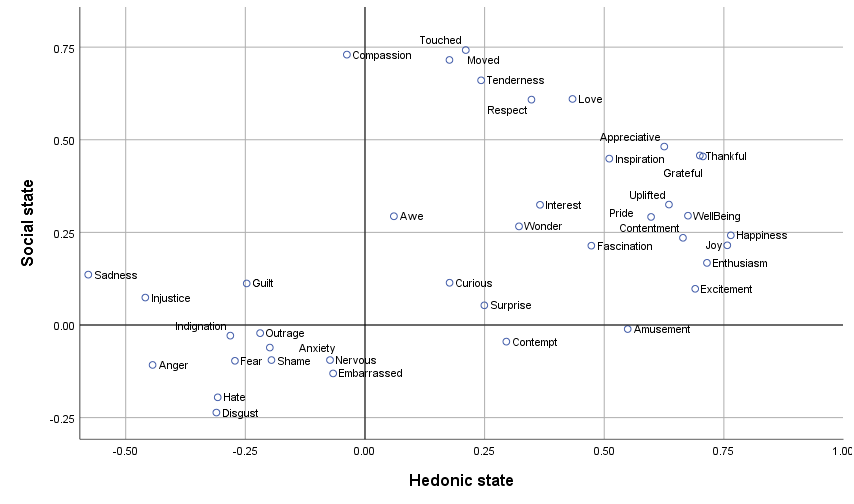
space divided by positive affect dimensions (Figure S1-3)

*Figure S1.* Pattern plot showing the correlations between the original variables of emotional labels and the first and second principal components extracted after the orthogonal rotation (first component on the *x*-axis and second component on the *y*-axis) in Study 1.


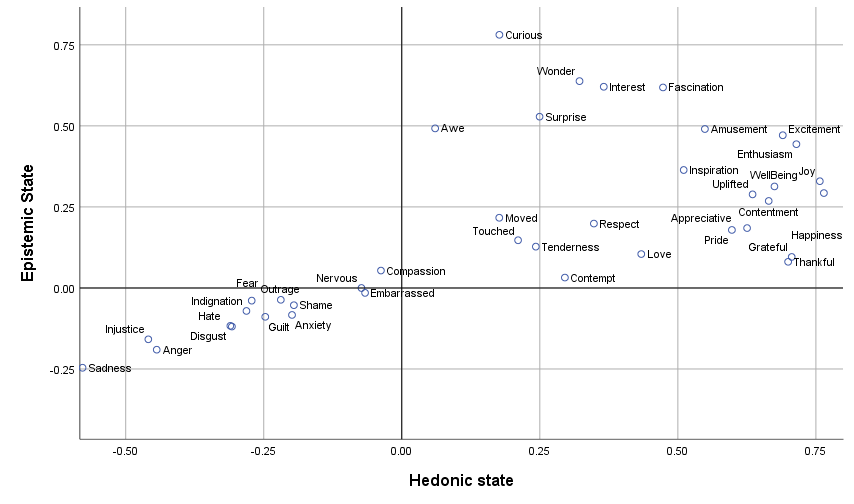


*Figure S2.* Pattern plot showing the correlations between the original variables of emotional labels and the first and fourth principal components extracted after the orthogonal rotation (first component on the *x*-axis and fourth component on the *y*-axis) in Study 1.


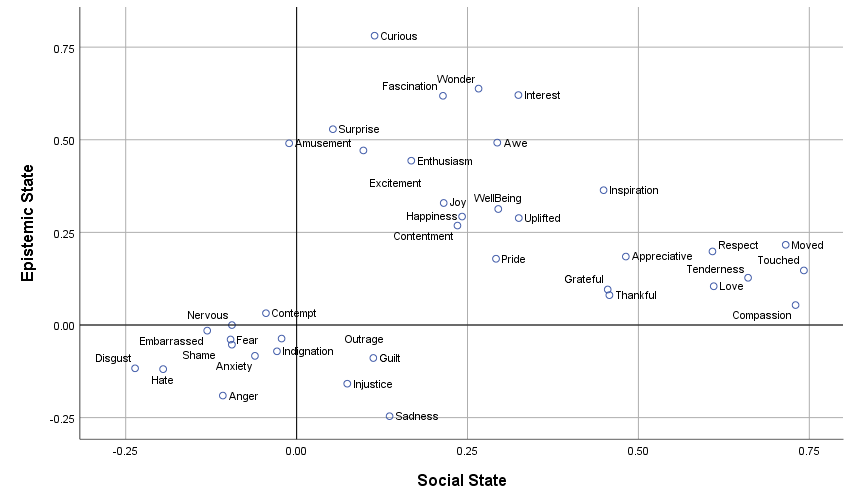


*Figure S3.* Pattern plot showing the correlations between the original variables of emotional labels and the second and fourth principal components extracted after the orthogonal rotation (second component on the *x*-axis and fourth component on the *y*-axis) in Study 1.

## Study 2

Instructions: “Think about the video you just watched. While watching the video, to which extent did you feel...” [Scale from 0 (not at all) to 6 (very strongly)]

We used the same list of emotional labels as in Study 1.

Results of Principal Component Analysis on 40 emotional labels representing the emotional space divided by positive affect dimensions (Figure S4-6)


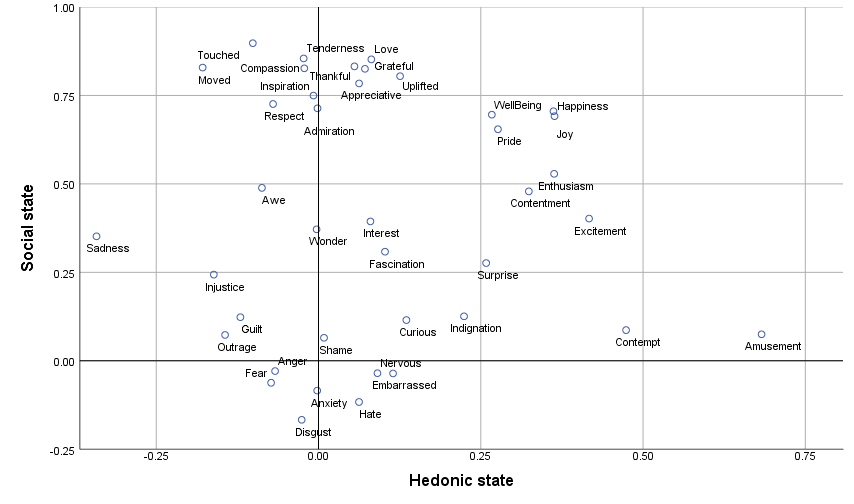


*Figure S4.* Pattern plot showing the correlations between the original variables of emotional labels and the fifth and first principal components extracted after the orthogonal rotation (fifth component on the *x*-axis and first component on the *y*-axis) in Study 2.


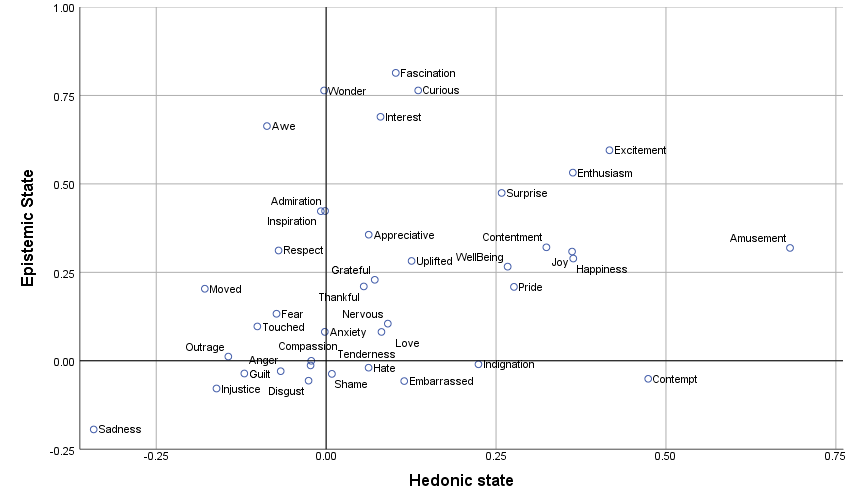


*Figure S5.* Pattern plot showing the correlations between the original variables of emotional labels and the fifth and second principal components extracted after the orthogonal rotation (fifth component on the *x*-axis and second component on the *y*-axis) in Study 2.


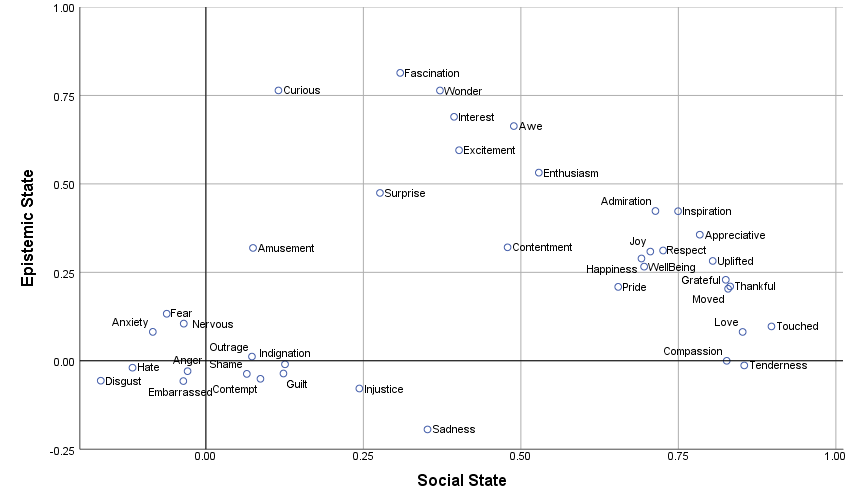


*Figure S6.* Pattern plot showing the correlations between the original variables of emotional labels and the first and second principal components extracted after the orthogonal rotation (first component on the *x*-axis and second component on the *y*-axis) in Study 2.

# Appendix C

**Basic Affective Dimensions**

## Study 1

*Basic Affective Dimensions*. Participants were asked to rate their experience at the time on five general dimensions: Valence, Control, Arousal, Impact and Pleasantness in order

1. Valence: Think of the situation you just described. How positive was this situation?
   - Scale from -5 (very negative) to 5 (very positive)
2. Control: Think of the situation you just described. To which extent did you feel you had control over the situation?
   - Scale from -5 (very little) to 5 (very much)
3. Arousal: Think of the situation you just described and the happiness you experienced at this time. How stimulating/arousing was this feeling?
   - Scale from -5 (very calm) to 5 (very stimulating/arousing)
4. Impact: Think of the situation you just described. How strong was the impact of the situation on you, your thoughts, and behaviours?
   - Scale from -5 (very weak) to 5 (very strong)
5. Pleasantness Think of what you experienced in the situation you just described. How pleasant or unpleasant was this experience?
   - Scale from -5 (very unpleasant) to 5 (very pleasant)

# Appendix D

**Bodily Feelings**

## Study 1

*Bodily feelings*. Participants were presented with 18 bodily feelings and asked to which extent they experienced them in the remembered situation (on a scale from 0 = “Not at all” to 6 = “Very strongly”). All items are presented in Table S3. The two distinct measures of Goosebumps (*r* = .77) were averaged into a unique measure.

Instructions:

“Please, indicate whether you experienced any of the following sensations or feelings in the remembered situation, and if so, to what extent.” [Scale from 0 (not at all) to 6 (very strongly)]

**Table S3**

*Items for bodily feelings, with their labels and the source they were drawn from.*

| **Item name** | **Question** | **Source** |
| --- | --- | --- |
| 1. *Blushing* | Blushing | Algoe & Haidt (2009) |
| 1. *Buoyant* | Buoyant or light | Algoe & Haidt (2009)  Zickfeld et al. (2019) |
| 1. *Chills* | Chills or shivers | Algoe & Haidt (2009)  Landmann, Cova & Hess (2019)  Yaden et al. (2019) |
| 1. *Chocked* | Chocked up | Zickfeld et al. (2019) |
| 1. *Goosebumps* | Goosebumps or hair standing up | Zickfeld et al. (2019) |
| 1. *Cold* | I felt cold |  |
| 1. *EyesWiden* | I felt my eyes widen | Yaden et al. (2019) |
| 1. *JawDrop* | I felt my jaw drop | Yaden et al. (2019) |
| 1. *Gasped* | I gasped | Yaden et al. (2019) |
| 1. *Goosebumps* | I had goosebumps | Landmann, Cova & Hess (2019)  Yaden et al. (2019) |
| 1. *HeartRate* | Increased heart rate | Algoe & Haidt (2009) |
| 1. *LumpThroat* | Lump in the throat | Algoe & Haidt (2009)  Zickfeld et al. (2019) |
| 1. *MoistEyes* | Moist eyes | Zickfeld et al. (2019) |
| 1. *MusclesRelaxed* | Muscles relaxed | Algoe & Haidt (2009) |
| 1. *MusclesTensed* | Muscles tensed | Algoe & Haidt (2009) |
| 1. *Refreshed* | Refreshed, energized or exhilarated | Zickfeld et al. (2019) |
| 1. *Tears* | Tears in my eyes | Algoe & Haidt (2009)  Landmann, Cova & Hess (2019)  Zickfeld et al. (2019) |
| 1. *WarmChest* | Warm feeling in the chest | Algoe & Haidt (2009)  Landmann, Cova & Hess (2019)  Zickfeld et al. (2019) |

A Principal Component Analysis with Varimax Rotation was conducted on the 17 items measuring bodily feelings. The number of retained components following the Kaiser’s method was three dimensions and the Scree Plot method fitted with this parsimonious choice. Despite this, the total variance explained by the three components is 55.2% and the three dimensions didn’t capture the most frequently reported bodily feelings that are usually attributed to transcendental emotions (Table S4). We forced the analysis to retain four, five and six dimensions to see which one gave us the cleanest factors structure and allowed us to investigate more nuances of bodily feelings. The solution in five dimensions, explaining 66.3% of the total variance, appeared to have the least number of cross-loading items, the most interpretable solution, and dimensions with three items at least (Costello & Osborne, 2005).

**Table S4**

*Solution in three dimensions of factor loadings in the rotated component matrix of Principal Component Analysis on items of bodily feelings. Bold: loadings >.5.*

|  | Component |  |  |
| --- | --- | --- | --- |
|  | 1 | 2 | 3 |
| 1. JawDrop | **.681** |  |  |
| 1. Goosebumps | **.653** |  |  |
| 1. Gasped | **.638** |  |  |
| 1. Chills | **.626** |  |  |
| 1. EyesWiden | **.619** | .355 |  |
| 1. HeartRate | **.612** |  | .328 |
| 1. MusclesTensed | **.608** | -.360 |  |
| 1. Cold | .486 | -.354 |  |
| 1. Refreshed |  | **.799** |  |
| 1. WarmChest |  | **.747** |  |
| 1. Buoyant |  | **.743** |  |
| 1. MusclesRelaxed |  | **.742** |  |
| 1. Blushing |  | .430 |  |
| 1. Tears |  |  | **.858** |
| 1. MoistEyes |  |  | **.847** |
| 1. LumpThroat | .341 |  | **.678** |
| 1. Chocked | .339 |  | **.612** |

**Table S5**

*Solution in five dimensions of the factor loadings in the rotated component matrix of Principal Component Analysis on items of bodily feelings. Bold: loadings >.50*

|  | Component |  |  |  |  |
| --- | --- | --- | --- | --- | --- |
|  | 1 | 2 | 3 | 4 | 5 |
| 1. Refreshed | **.785** |  |  |  |  |
| 1. Muscles Relaxed | **.779** |  |  |  |  |
| 1. Buoyant | **.749** |  |  |  |  |
| 1. Warm Chest | **.729** |  |  |  |  |
| 1. Tears |  | **.874** |  |  |  |
| 1. Moist Eyes |  | **.861** |  |  |  |
| 1. Lump Throat |  | **.638** |  | .326 |  |
| 1. Choked |  | **.610** |  |  |  |
| 1. Jaw Drop |  |  | **.803** |  |  |
| 1. Gasped |  |  | **.767** |  |  |
| 1. Eyes Widen |  |  | **.675** |  |  |
| 1. Chills |  |  |  | **.780** |  |
| 1. Cold |  |  |  | **.699** |  |
| 1. Goosebumps |  |  |  | **.637** |  |
| 1. Heart Rate |  |  |  |  | **.683** |
| 1. Blushing | **.**402 |  |  |  | **.650** |
| 1. Muscles Tensed | -.359 |  |  | **.**407 | **.557** |

The solution in five dimensions seems to capture five well-defined different bodily feelings (Table S5). As for emotional labels, we considered loadings above .50 and ignored loading below .32 (Tabachnick & Fidell, 2013). The first general component, explaining 30% of total variance, is measured by items describing positive and calm bodily feelings and could be structuring all the answers related to a “Relax”. The second component, explaining 18.1 % of the total variance, stems from items describing bodily feelings related to “Crying”, the third one, explaining 8.1% of the total variance, is composed by items related to a “Shock”, the fourth component, explaining 6% of the variance is composed by items related to “Chills”, and the fifth, explaining 5.3% of the variance, describes bodily feelings typical of “Activation”.

## Study 2

*Bodily feelings*. Participants were presented with 17 bodily reactions/bodily feelings and asked to which extent they experienced them while watching the video-clip (on a scale from 0 = “Not at all” to 6 = “Very strongly”). All items are the same as in study 1 and are presented in Table S6, but one of the measures of Goosebumps (“Goosebumps or hair standing up”) was dropped because it was redundant.

Instructions:

“To which extent did you experience the following feelings or had the following sensations or feelings while watching the part of the video you remember more vividly?” [Scale from 0 (not at all) to 6 (very strongly)]

**Table S6**

*Items for bodily feelings, with their labels and the source they were drawn from.*

| **Item name** | **Question** | **Source** |
| --- | --- | --- |
| 1. *Blushing* | Blushing | Algoe & Haidt (2009) |
| 1. *Buoyant* | Buoyant or light | Algoe & Haidt (2009)  Zickfeld et al. (2019) |
| 1. *Chills* | Chills or shivers | Algoe & Haidt (2009)  Landmann, Cova & Hess (2019)  Yaden et al. (2019) |
| 1. *Chocked* | Chocked up | Zickfeld et al. (2019) |
| 1. *Cold* | I felt cold |  |
| 1. *EyesWiden* | I felt my eyes widen | Yaden et al. (2019) |
| 1. *JawDrop* | I felt my jaw drop | Yaden et al. (2019) |
| 1. *Gasped* | I gasped | Yaden et al. (2019) |
| 1. *Goosebumps* | I had goosebumps | Landmann, Cova & Hess (2019)  Yaden et al. (2019) |
| 1. *HeartRate* | Increased heart rate | Algoe & Haidt (2009) |
| 1. *LumpThroat* | Lump in the throat | Algoe & Haidt (2009)  Zickfeld et al. (2019) |
| 1. *MoistEyes* | Moist eyes | Zickfeld et al. (2019) |
| 1. *MusclesRelaxed* | Muscles relaxed | Algoe & Haidt (2009) |
| 1. *MusclesTensed* | Muscles tensed | Algoe & Haidt (2009) |
| 1. *Refreshed* | Refreshed, energized or exhilarated | Zickfeld et al. (2019) |
| 1. *Tears* | Tears in my eyes | Algoe & Haidt (2009)  Landmann, Cova & Hess (2019)  Zickfeld et al. (2019) |
| 1. *WarmChest* | Warm feeling in the chest | Algoe & Haidt (2009)  Landmann, Cova & Hess (2019)  Zickfeld et al. (2019) |

As in study 1, a Principal Component Analysis with Varimax Rotation was conducted on the 17 items measuring the physiological states. The number of retained components following the Kaiser’s method was three dimensions and the Scree Plot method fitted with this parsimonious choice. We forced the analysis to retain four, five and six dimensions to see which one gave us the cleanest factor structure and allowed us to investigate more nuances of physiological states. The solution in five dimensions, explaining 73,5% of the total variance, didn’t fit the criteria cited by Costello and Osborne(Costello & Osborne, 2005) because it appeared to have more cross-loading items than the three-dimension solution, and because only two items loaded on the fifth dimension, making it unstable. Despite the concerns about consistency between the two studies, we decided to retain the solution in three dimensions (Table S7).

**Table S7**

*Solution in three dimensions of the factor loadings in the rotated component matrix of Principal Component Analysis on items of physiological states (study 2). Bold: loadings >.50. Italics: Items without any loading >.5.*

|  | Component | |  |
| --- | --- | --- | --- |
|  | 1 | 2 | 3 |
| 1. HeartRate | **.728** |  |  |
| 1. MusclesTensed | **.726** |  |  |
| 1. JawDrop | **.723** |  |  |
| 1. Gasped | **.713** |  |  |
| 1. Goosebumps | **.652** | .388 |  |
| 1. Chills | **.64** | .378 |  |
| 1. EyesWiden | **.614** |  | .474 |
| 1. Cold | **.542** |  |  |
| 1. *Blushing* | .438 |  |  |
| 1. Tears |  | **.900** |  |
| 1. MoistEyes |  | **.896** |  |
| 1. Chocked |  | **.858** |  |
| 1. LumpThroat | .340 | **.793** |  |
| 1. Refreshed |  |  | **.805** |
| 1. MusclesRelaxed |  |  | **.769** |
| 1. Buoyant |  |  | **.741** |
| 1. WarmChest |  | **.505** | **.613** |

The total variance explained by the three components is 63,9 % and the three dimensions captured very easy distinctive bodily feelings. Again, we considered loadings above .50 and ignored loading below .32 (Tabachnick & Fidell, 2013). The first general component, explaining 41.3% of total variance, is measured by items describing activated and alerted bodily feelings and could be structuring all the answers related to a “Activation”. The second component, explaining 12.4 % of the total variance, stems from items describing bodily feelings related to “Crying”, the third one, explaining 10.2% of the total variance, is composed by items describing positive and calm bodily feeling related to “Relax”.

# Appendix E

**Cognitive and situation appraisals**

## Study 1

*Cognitive appraisals*. Participants were presented with 44 items probing their cognitive appraisal of the situation and asked to which extent they had the following feelings or thoughts in the remembered situation (on a scale from 0 = “Not at all” to 6 = “Very strongly”). All items are presented in Table S8.

Instructions:

“To which extent did you experience the following feelings or had the following thoughts in the situation you described?” [Scale from 0 (not at all) to 6 (very strongly)]

**Table S8**

*Items for cognitive appraisals, with their labels and the source they were drawn from (study 1).*

| **Item name** | **Question** | **Source** |
| --- | --- | --- |
| 1. *ReducedSelf* | I experienced a reduced sense of the self | Yaden et al., (2019) |
| 1. *SomethingGreater* | I experienced something greater than myself | Shiota et al., (2007); Yaden et al. (2019) |
| 1. *PassageTime* | I experienced the passage of time differently | Yaden et al. (2019) |
| 1. *Closeness* | I felt an exceptional sense of closeness | Zickfeld et al. (2019) |
| 1. *Welcomed* | I felt an extraordinary feeling of welcoming or being welcomed | Zickfeld et al. (2019) |
| 1. *IncredibleBond* | I felt an incredible bond | Zickfeld et al. (2019) |
| 1. *UniqueLove* | I felt a unique kind of love spring up | Zickfeld et al. (2019) |
| 1. *Communion* | I felt a sense of communion with all living things | Yaden et al. (2019) |
| 1. *MentalChallenged* | I felt challenged to mentally process what I was experiencing | Yaden et al. (2019) |
| 1. *UnderstandChallenged* | I felt challenged to understand the experience | Yaden et al. (2019) |
| 1. *Humanity* | I felt closely connected to humanity | Yaden et al. (2019) |
| 1. *GrandScheme* | I felt insignificant in the grand scheme of things | Piff et al. (2015) |
| 1. *HelpOther* | I felt like I needed to do more to help other people |  |
| 1. *SomethingGrand* | I felt like I was in presence of something grand | Huta & Ryan, (2010); Piff et al. (2015); Yaden et al., 2019 |
| 1. *Deep* | I felt like I was learning something deep and/or profound |  |
| 1. *GreaterWhole* | I felt like I was part of a greater whole | Huta & Ryan (2010); Piff et al. (2015) |
| 1. *NoMatter* | I felt like, in the grand scheme of things, my own issues and concerns did not matter as much | Piff et al. (2015) |
| 1. *Meaningful* | I felt like my life was meaningful |  |
| 1. *Meaningless* | I felt like my life was meaningless |  |
| 1. *Trivial* | I felt like my own day-to-day concerns were relatively trivial | Piff et al. (2015) |
| 1. *Commitment* | I felt more strongly committed to a relationship | Zickfeld et al. (2019) |
| 1. *Smaller* | I felt my sense of self become somehow smaller | Yaden et al. (2019) |
| 1. *Optimistic* | I felt optimistic about humanity |  |
| 1. *GreaterEntity* | I felt part of some greater entity | Huta & Ryan (2010); Piff et al. (2015) |
| 1. *Shrink* | I felt my sense of self shrink | Yaden et al. (2019) |
| 1. *TimeChange* | I felt my sense of time change | Yaden et al. (2019) |
| 1. *Insignificant* | I felt small or insignificant | Shiota, et al. (2007); Piff et al. (2015) |
| 1. *MorePowerful* | I felt small relative to something more powerful than myself | Piff et al. (2015) |
| 1. *Diminished* | I felt that my sense of self was diminished | Yaden et al. (2019) |
| 1. *PowerfulThings* | I felt the existence of things more powerful than myself | Piff et al. (2015) |
| 1. *GreaterThanMyself* | I felt the presence of something greater than myself | Shiota, et al. (2007); Piff et al. (2015) |
| 1. *HardToComprehend* | I found it hard to comprehend the experience in full | Yaden et al. (2019) |
| 1. *Connectedness* | I had a sense of complete connectedness | Yaden et al. (2019) |
| 1. *PeopleAreGood* | I had the feeling that people were really good | Aquino et al., (2011) |
| 1. *StillSomeGood* | I had the feeling that there was still some good in this world | Aquino et al., (2011) |
| 1. *Kindness* | I had the feeling that the world was full of kindness and generosity | Aquino et al., (2011) |
| 1. *AdmirableActions* | I had the feeling that the actions of most people were admirable | Aquino et al., (2011) |
| 1. *ConnectedToEverything* | I had the sense of being connected to everything | Yaden et al. (2019) |
| 1. *LastedLonger* | I had the sense that a moment lasted longer than usual | Yaden et al. (2019) |
| 1. *TimeSlowing* | I noticed time slowing | Yaden et al. (2019) |
| 1. *Vastness* | I perceived vastness | Yaden et al. (2019) |
| 1. *SlowDown* | I sensed things momentarily slow down | Yaden et al. (2019) |
| 1. *Struggle* | I struggled to take in all that I was experiencing at once | Yaden et al. (2019) |
| 1. *Magnitude* | I tried to understand the magnitude of what I was experiencing | Yaden et al. (2019) |

*Situation appraisals*. Finally, participants were presented with 14 statements about the situation they remembered and asked to rate their agreement with each of these statements (on a scale from -3 = “Fully disagree” to 3 = “Full agree”). All items are presented in Table S9.

Instructions:

“To which extent do you agree with the following statements about the situation you described?” [Scale from -3 (Fully disagree) to 3 (Full agree)]

**Table S9**

*Items for situation appraisals, with their labels and the source they were drawn from.*

| **Item name** | **Question** | **Source** |
| --- | --- | --- |
| 1. *Adorable* | It was adorable |  |
| 1. *Meaningful* | It was a meaningful experience |  |
| 1. *Cute* | It was cute |  |
| 1. *Heartwarming* | It was heartwarming | Zickfeld et al. (2019) |
| 1. *Unfair* | It was unfair |  |
| 1. *Surprising* | It was surprising |  |
| 1. *Terrifying* | It was terrifying |  |
| 1. *Outraging* | It was outraging |  |
| 1. *Bonds* | The situation featured strong bonds between people | Landmann, Cova & Hess (2019) |
| 1. *BetterNature* | The situation exemplified humanity's better nature | Landmann, Cova & Hess (2019) |
| 1. *MoralBehavior* | The situation featured morally and ethically praiseworthy behavior | Landmann, Cova & Hess (2019) |
| 1. *GoodPerformance* | The situation featured a remarkably good performance from someone else | Landmann, Cova & Hess (2019) |
| 1. *Beyond* | The situation showed how people can go beyond themselves | Landmann, Cova & Hess (2019) |
| 1. *Surpassing* | The situation showed someone surpassing all expectations | Landmann, Cova & Hess (2019) |

A Principal Component Analysis (PCA) with Varimax Rotation was conducted on the 44 items measuring the cognitive appraisals and the 14 items measuring the situation appraisals. The two measures were separated in the questionnaire because they referred to different sides of the experience but were merged in the analysis because both are part of the cognitive evaluation of the relation between the self and the environment. Also, separating or merging the two scales do not change the component extracted by the PCA. The number of retained components following the Kaiser’s method was 9, with the last component being measured only by 2 variables with loadings <.65 (“Trivial” and “No Matter”). The Scree Plot method does not give a clear proposal, but it seems to suggest retaining 5 dimensions, which is a far too costly solution in terms of precision of the information extracted. Velicer’s MAP criteria (Zwick & Velicer, 1986) suggests to retain 8 dimensions. The PCA was forced to retain 8 components (see Table S10) and the total variance explained by those is 65%.

**Table S10**

*Factor loadings in the rotated component matrix of Principal Component Analysis on items of cognitive and situation appraisals. Bold: loadings >.50, Italics: Items without any loading >.5.*

|  | Component | | | | | | | |
| --- | --- | --- | --- | --- | --- | --- | --- | --- |
|  | 1 | 2 | 3 | 4 | 5 | 6 | 7 | 8 |
| 1. Greater Than Myself | **.780** |  |  |  |  |  |  |  |
| 1. Powerful Things | **.746** |  |  |  |  |  |  |  |
| 1. Something Greater | **.746** |  |  |  |  |  |  |  |
| 1. Something Grand | **.728** |  |  |  |  |  |  |  |
| 1. Greater Entity | **.723** |  |  |  |  |  |  |  |
| 1. Greater Whole | **.712** |  |  |  |  |  |  |  |
| 1. Connected To Everything | **.655** |  |  |  |  |  |  |  |
| 1. More Powerful | **.596** |  | .412 |  |  |  |  |  |
| 1. Vastness | **.596** |  |  |  |  |  |  |  |
| 1. Communion | **.595** |  |  |  |  |  |  |  |
| 1. Deep | **.512** |  |  |  |  |  |  |  |
| 1. *No Matter* | .488 |  | .334 |  |  |  |  |  |
| 1. *Humanity* | .472 | .347 |  |  |  |  |  | .449 |
| 1. *Trivial* | .419 |  |  |  |  |  |  |  |
| 1. Situation_Beyond |  | **.834** |  |  |  |  |  |  |
| 1. Situation_Good Performance |  | **.820** |  |  |  |  |  |  |
| 1. Situation_Surpassing |  | **.791** |  |  |  |  |  |  |
| 1. Situation_Moral Behavior |  | **.790** |  |  |  |  |  |  |
| 1. Situation_Better Nature |  | **.743** |  |  |  |  |  |  |
| 1. Diminished |  |  | **.790** |  |  |  |  |  |
| 1. Shrink |  |  | **.776** |  |  |  |  |  |
| 1. Smaller |  |  | **.776** |  |  |  |  |  |
| 1. Reduced Self |  |  | **.749** |  |  |  |  |  |
| 1. Insignificant |  |  | **.715** |  |  |  |  |  |
| 1. Meaningless |  |  | **.669** |  |  |  |  |  |
| 1. Grand Scheme | .354 |  | **.644** |  |  |  |  |  |
| 1. Situation_Unfair |  |  |  | **-.772** |  |  |  |  |
| 1. Situation_Adorable |  |  |  | **.738** | .344 |  |  |  |
| 1. Situation_Outraging |  |  |  | **-.724** |  |  |  |  |
| 1. Situation_Cute |  |  |  | **.706** | .369 |  |  |  |
| 1. Situation_Terrifying |  |  |  | **-.695** |  |  |  |  |
| 1. Situation_Heart Warming |  | .390 |  | **.668** |  |  |  |  |
| 1. *Situation_Meaningful* |  | .346 |  | .384 |  |  |  |  |
| 1. Commitment |  |  |  |  | **.716** |  |  |  |
| 1. Incredible Bond | .328 |  |  |  | **.713** |  |  |  |
| 1. Closeness |  |  |  |  | **.687** |  |  |  |
| 1. Unique Love | .348 |  |  | .347 | **.597** |  |  |  |
| 1. Situation_Bonds |  | **.574** |  |  | **.578** |  |  |  |
| 1. Connectedness | .482 |  |  |  | **.529** |  |  |  |
| 1. *Welcomed* |  |  |  | .423 | .448 |  |  |  |
| 1. *Meaningful* | .385 |  |  |  | .408 |  |  |  |
| 1. Time Slowing |  |  |  |  |  | **.822** |  |  |
| 1. Slow Down |  |  |  |  |  | **.761** |  |  |
| 1. Passage Time |  |  |  |  |  | **.760** |  |  |
| 1. Lasted Longer |  |  |  |  |  | **.742** |  |  |
| 1. Time Change |  |  |  |  |  | **.731** |  |  |
| 1. Mental Challenged |  |  |  |  |  |  | **.701** |  |
| 1. Understand Challenged |  |  |  |  |  |  | **.677** |  |
| 1. Struggle |  |  |  |  |  |  | **.662** |  |
| 1. Hard To Comprehend |  |  |  |  |  |  | **.634** |  |
| 1. Magnitude | .416 |  |  |  |  |  | **.578** |  |
| 1. *Situation_Surprising* |  |  |  |  |  |  | .350 |  |
| 1. Optimistic |  | .415 |  | .387 |  |  |  | **.541** |
| 1. People Are Good |  | .**499** |  |  |  |  |  | **.534** |
| 1. Kindness |  | .386 |  | .398 |  |  |  | **.519** |
| 1. Admirable Actions |  | .493 |  |  |  |  |  | **.505** |
| 1. Help Other |  | .412 |  |  |  |  |  | **.497** |
| 1. *Still Some Good* |  | .415 |  | .399 |  |  |  | .458 |

**Table S11**

*Communality values of cognitive evaluations items before and after extraction of eight dimensions, representing the variance of each item that is predictable from the extracted dimensions.*

|  | Communalities | |
| --- | --- | --- |
|  | Initial | Extraction |
| ReducedSelf | 1.000 | .657 |
| SomethingGreater | 1.000 | .702 |
| PassageTime | 1.000 | .722 |
| Closeness | 1.000 | .698 |
| Welcomed | 1.000 | .645 |
| IncredibleBond | 1.000 | .752 |
| UniqueLove | 1.000 | .707 |
| Communion | 1.000 | .587 |
| MentalChallenged | 1.000 | .662 |
| UnderstandChallenged | 1.000 | .617 |
| Humanity | 1.000 | .686 |
| GrandScheme | 1.000 | .598 |
| Help Other | 1.000 | .547 |
| Something Grand | 1.000 | .685 |
| Deep | 1.000 | .540 |
| Greater Whole | 1.000 | .678 |
| NoMatter | 1.000 | .427 |
| Meaningful | 1.000 | .535 |
| Meaningless | 1.000 | .521 |
| Trivial | 1.000 | .395 |
| Commitment | 1.000 | .642 |
| Smaller | 1.000 | .703 |
| Optimistic | 1.000 | .755 |
| Greater Entity | 1.000 | .664 |
| Shrink | 1.000 | .685 |
| Time Change | 1.000 | .703 |
| Insignificant | 1.000 | .670 |
| More Powerful | 1.000 | .593 |
| Diminished | 1.000 | .695 |
| Powerful Things | 1.000 | .678 |
| Greater Than Myself | 1.000 | .728 |
| Hard To Comprehend | 1.000 | .570 |
| Connectedness | 1.000 | .631 |
| People Are Good | 1.000 | .757 |
| Still Some Good | 1.000 | .694 |
| Kindness | 1.000 | .750 |
| Admirable Actions | 1.000 | .666 |
| Connected To Everything | 1.000 | .674 |
| Lasted Longer | 1.000 | .668 |
| Time Slowing | 1.000 | .786 |
| Vastness | 1.000 | .521 |
| Slow Down | 1.000 | .712 |
| Struggle | 1.000 | .599 |
| Magnitude | 1.000 | .597 |
| Situation_Adorable | 1.000 | .777 |
| Situation_Meaningful | 1.000 | .451 |
| Situation_Cute | 1.000 | .743 |
| Situation_Heartwarming | 1.000 | .759 |
| Situation_Unfair | 1.000 | .708 |
| Situation_Surprising | 1.000 | .288 |
| Situation_Terrifying | 1.000 | .632 |
| Situation_Outraging | 1.000 | .629 |
| Situation_Bonds | 1.000 | .670 |
| Situation_Better Nature | 1.000 | .718 |
| Situation_Moral Behavior | 1.000 | .729 |
| Situation_GoodPerformance | 1.000 | .756 |
| Situation_Beyond | 1.000 | .741 |
| Situation_Surpassing | 1.000 | .696 |

The first component, explaining 14 % of the total variance of the items, seems to capture a dimension of being “Within Some Greater”. The second one, explaining 10% of the total variance of the items, seems to capture “Display of Outstanding Standard” evaluation and the third dimension, explaining 8.9% of the total variance, seems to capture the perception of a “Small Self”. The fourth principal component, explaining 8.8% of the variance, seem to assemble the items about the “Situational Valence”. This is a bipolar component, while the others are all unipolar. The fifth, explaining 7.3% of the total variance, “Feeling of Social Connection” evaluation, the sixth, explaining 6.4% of the total variance, seems to capture the “Time Perception”, the seventh explains 5.3% of the total variance and seems to capture the “Mental Challenge” perception and the last explains 4.4% of the total variance and seems to capture the feeling of “Optimism about Humanity” (Table S10). Some items’ specificities as variables measuring one singular component is not very clear: Welcomed, Meaningful, and Still Some Good do not reach >.50 loadings on one specific component. The variance of some items is not well captured by the extracted components and those items (No Matter, Trivial, Situation_Meaningful, Situation_Surprising) have low extracted communality values, *h*^2^ <.5 (Table S11). As mentioned above, adding one dimension creates one component capturing the variance of the items Trivial and No Matter, but it does not change loadings and communalities of other items. Also, the number of cross-loadings is not surprising, because the cognitive evaluation items were associated to positive emotions and specifically the items were cognitive evaluations specifically related to transcendent emotions. More importantly, to make sense of the extracted dimensions and name them, all the loadings <.32 were ignored.

## Study 2

*Cognitive appraisals*. Participants were presented with 48 items probing their cognitive appraisal of the situation and asked to which extent they had the following feelings or thoughts while watching the video-clip (on a scale from 0 = “Not at all” to 6 = “Very strongly”). All items are presented in Table S12.

On the basis of the results of Study 1, we decided to drop six items that didn’t load on any dimension, and we decided to add some items about the perceived meaningfulness of the situation.

Instructions:

“To which did you experience the following feelings or had the following thoughts while watching the part of the video you remember more vividly?” [Scale from 0 (not at all) to 6 (very strongly)]

**Table S12**

*Items for cognitive appraisals, with their labels and the source they were drawn from (study 2). Bold characters are for items that has been added in this study and were not present in Study 1.*

| **Item Name** | **Question** | **Source** |
| --- | --- | --- |
| 1. *ReducedSelf* | I experienced a reduced sense of the self | Yaden et al. (2019) |
| 1. *SomethingGreater* | I experienced something greater than myself | Shiota, et al. (2007); Yaden et al. (2019) |
| 1. *PassageTime* | I experienced the passage of time differently | Yaden et al. (2019) |
| 1. *Closeness* | I felt an exceptional sense of closeness | Zickfeld et al. (2019) |
| 1. *IncredibleBond* | I felt an incredible bond | Zickfeld et al. (2019) |
| 1. *UniqueLove* | I felt a unique kind of love spring up | Zickfeld et al. (2019) |
| 1. *Communion* | I felt a sense of communion with all living things | Yaden et al. (2019) |
| 1. *MentalChallenged* | I felt challenged to mentally process what I was experiencing | Yaden et al. (2019) |
| 1. *UnderstandChallenged* | I felt challenged to understand the experience | Yaden et al. (2019) |
| 1. *HelpOther* | I felt like I needed to do more to help other people |  |
| 1. *SomethingGrand* | I felt like I was in presence of something grand | Huta & Ryan (2010); Piff et al. (2015); Yaden et al., 2019 |
| 1. *Deep* | I felt like I was learning something deep and/or profound |  |
| 1. *GreaterWhole* | I felt like I was part of a greater whole | Huta & Ryan (2010); Piff et al. (2015) |
| 1. *Meaningful* | I felt like my life was meaningful |  |
| 1. *Meaningless* | I felt like my life was meaningless |  |
| 1. *Commitment* | I felt more strongly committed to a relationship | Zickfeld et al. (2019) |
| 1. *Smaller* | I felt my sense of self become somehow smaller | Yaden et al. (2019) |
| 1. *Optimistic* | I felt optimistic about humanity |  |
| 1. *GreaterEntity* | I felt part of some greater entity | Huta & Ryan (2010); Piff et al. (2015) |
| 1. *Shrink* | I felt my sense of self shrink | Yaden et al. (2019) |
| 1. *TimeChange* | I felt my sense of time change | Yaden et al. (2019) |
| 1. *Insignificant* | I felt small or insignificant | Shiota, et al. (2007); Piff et al. (2015) |
| 1. *MorePowerful* | I felt small relative to something more powerful than myself | Piff et al. (2015) |
| 1. *Diminished* | I felt that my sense of self was diminished | Yaden et al. (2019) |
| 1. *PowerfulThings* | I felt the existence of things more powerful than myself | Piff et al. (2015) |
| 1. *GreaterThanMyself* | I felt the presence of something greater than myself | Shiota, et al. (2007); Piff et al. (2015) |
| 1. *HardToComprehend* | I found it hard to comprehend the experience in full | Yaden et al. (2019) |
| 1. *Connectedness* | I had a sense of complete connectedness | Yaden et al. (2019) |
| 1. *PeopleAreGood* | I had the feeling that people were really good | Aquino et al., (2011) |
| 1. *Kindness* | I had the feeling that the world was full of kindness and generosity | Aquino et al., (2011) |
| 1. *AdmirableActions* | I had the feeling that the actions of most people were admirable | Aquino et al., (2011) |
| 1. *ConnectedToEverything* | I had the sense of being connected to everything | Yaden et al. (2019) |
| 1. *LastedLonger* | I had the sense that a moment lasted longer than usual | Yaden et al. (2019) |
| 1. *TimeSlowing* | I noticed time slowing | Yaden et al. (2019) |
| 1. *Vastness* | I perceived vastness | Yaden et al. (2019) |
| 1. *SlowDown* | I sensed things momentarily slow down | Yaden et al. (2019) |
| 1. *Struggle* | I struggled to take in all that I was experiencing at once | Yaden et al. (2019) |
| 1. *Magnitude* | I tried to understand the magnitude of what I was experiencing | Yaden et al. (2019) |
| 1. ***BeyondMyself*** | I realized that I should positively contribute to something beyond myself. |  |
| 1. ***MoreThanPleasure*** | I felt like there is something more than mere pleasure in life. |  |
| 1. ***SomethingWorthwhile*** | I felt like there was something worthwhile in life. |  |
| 1. ***TimeMatter*** | I felt like I should spend more time on things that really matter. |  |
| 1. ***WorthFighting*** | I felt like there were things worth fighting for. |  |
| 1. ***ReallyImportant*** | I felt like I knew what was really important. |  |
| 1. ***UnderstoodWhatMatters*** | I felt like I finally understood what mattered in life. |  |
| 1. ***RevisingPriorities*** | I felt like revising my own priorities |  |
| 1. ***ImproveMyself*** | I wanted to improve myself |  |
| 1. ***BetterPerson*** | I felt like becoming a better person |  |

*Situation appraisals*. Finally, participants were presented with six statements about the video they just watched and asked to rate their agreement with each of these statements (on a scale from -3 = “Fully disagree” to 3 = “Full agree”). All items are presented in Table S13.

Instructions:

“To which extent do you agree with the following statements about the video you just watched?” [Scale from -3 (Fully disagree) to 6 (Full agree)]

**Table S13**

*Items for situation appraisals, with their labels and the source they were drawn from.*

| **Item name** | **Question** | **Source** |
| --- | --- | --- |
| 1. *MeaningfulSituation* | The situation taught me something meaningful |  |
| 1. *BetterNature* | The situation exemplified humanity's better nature | Landmann, Cova & Hess (2019) |
| 1. *MoralBehavior* | The situation featured morally and ethically praiseworthy behavior | Landmann, Cova & Hess (2019) |
| 1. *GoodPerformance* | The situation featured a remarkably good performance from someone else | Landmann, Cova & Hess (2019) |
| 1. *Beyond* | The situation showed how people can go beyond themselves | Landmann, Cova & Hess (2019) |
| 1. *Surpassing* | The situation showed someone surpassing all expectations | Landmann, Cova & Hess (2019) |

As in Study 1, a Principal Component Analysis (PCA) with Varimax Rotation was conducted on the 48 items measuring the cognitive appraisals and the six items measuring the situation appraisals. The two measures were separated in the questionnaire because they referred to different sides of the emotional experience but were merged in the analysis because both are part of the cognitive evaluation of the relation between the self and the environment. Also, separating or merging the two scales do not change the component extracted by the PCA.

The number of retained components following the Kaiser’s method was six, and the Scree Plot method suggested to retain three dimensions, which is a far too costly solution in terms of precision of the information extracted. We forced the analysis to retain seven and eight dimensions to see which choice could explain the most amount of variance preserving a stable structure of dimensions. The solutions in seven and eight dimensions, explaining 76.4% and 77.6% of the total variance, added dimensions composed by less than three items and loadings <.0.50 and were considered solutions with unstable dimensions. We decided to retain six components (see Table S14) and the total variance explained by those is 74.9%.

Even if our aim was to create a dimension of meaningfulness, using items related to the cognitive evaluation of a meaningful situation, the PCA showed that the dimension of meaningfulness was not distinguished from the evaluation of “Feeling of Social Connection”. This is in accordance with findings that show that the most cited source of meaningfulness in life is social connection (Schnell, 2020).

**Table S14**

*Factor loadings in the rotated component matrix of Principal Component Analysis on items of cognitive and situation appraisals. Bold: loadings >.50, Italics: Items without any loading >.5.*

|  | | | | |  |  |
| --- | --- | --- | --- | --- | --- | --- |
|  | Component | | | | | |
|  | 1 | 2 | 3 | 4 | 5 | 6 |
| 1. BeyondMyself | **.803** |  |  |  |  |  |
| 1. BetterPerson | **.801** |  |  |  |  |  |
| 1. ImproveMyself | **.785** |  |  |  |  |  |
| 1. TimeMatter | **.78** |  |  |  |  |  |
| 1. WorthFighting | **.764** |  |  |  |  |  |
| 1. HelpOther | **.76** |  |  |  |  |  |
| 1. ReallyImportant | **.756** |  |  |  |  |  |
| 1. UnderstoodWhatMatters | **.737** |  |  |  |  |  |
| 1. SomethingWorthwhile | **.731** | .356 |  |  |  |  |
| 1. Meaningful | **.722** | .326 |  |  |  |  |
| 1. RevisingPriorities | **.721** |  |  | .351 |  |  |
| 1. Kindness | **.715** |  | .392 |  |  |  |
| 1. UniqueLove | **.707** |  |  |  |  |  |
| 1. Commitment | **.703** |  |  |  |  |  |
| 1. Closeness | **.698** |  |  |  |  |  |
| 1. MoreThanPleasure | **.698** | .322 |  |  |  |  |
| 1. Optimistic | **.698** |  | .43 |  |  |  |
| 1. PeopleAreGood | **.695** |  | .47 |  |  |  |
| 1. IncredibleBond | **.688** | .354 |  |  |  |  |
| 1. AdmirableActions | **.678** |  | .409 |  |  |  |
| 1. Connectedness | **.672** | .4 |  |  |  |  |
| 1. Communion | **.645** | .465 |  |  |  |  |
| 1. ConnectedToEverything | **.635** | .477 |  |  |  |  |
| 1. GreaterWhole | **.613** | **.555** |  |  |  |  |
| 1. Deep | **.58** | .419 |  |  |  |  |
| 1. PowerfulThings | **.496** | **.636** |  |  |  |  |
| 1. GreaterThanMyself | **.505** | **.629** |  |  |  |  |
| 1. Vastness |  | **.618** |  | .336 |  |  |
| 1. SomethingGrand | .478 | **.614** |  |  |  |  |
| 1. SomethingGreater | **.524** | **.608** |  |  |  |  |
| 1. GreaterEntity | **.55** | **.6** |  |  |  |  |
| 1. MorePowerful |  | **.591** |  | .472 |  |  |
| 1. Beyond | .376 |  | **.835** |  |  |  |
| 1. MoralBehavior | .375 |  | **.825** |  |  |  |
| 1. Surpassing | .368 |  | **.821** |  |  |  |
| 1. GoodPerformance |  |  | **.81** |  |  |  |
| 1. BetterNature | .425 |  | **.775** |  |  |  |
| 1. MeaningfulSituation | **.505** |  | **.527** |  |  |  |
| 1. Shrink |  |  |  | **.769** |  |  |
| 1. Diminished |  |  |  | **.764** |  |  |
| 1. Insignificant |  | .354 |  | **.727** |  |  |
| 1. Smaller |  |  |  | **.723** |  |  |
| 1. ReducedSelf |  |  |  | **.718** |  |  |
| 1. Meaningless |  |  |  | **.686** |  |  |
| 1. TimeSlowing |  |  |  |  | **.832** |  |
| 1. SlowDown |  |  |  |  | **.783** |  |
| 1. TimeChange |  |  |  |  | **.767** |  |
| 1. PassageTime |  |  |  |  | **.758** |  |
| 1. LastedLonger | .357 |  |  |  | **.709** |  |
| 1. MentalChallenged |  |  |  |  |  | **.769** |
| 1. UnderstandChallenged |  |  |  |  |  | **.707** |
| 1. HardToComprehend |  |  |  | .369 |  | **.684** |
| 1. Struggle |  |  |  | .394 |  | **.632** |
| 1. *Magnitude* | .389 | .426 |  |  |  | .458 |

The first component, explaining 52.8 % of the total variance of the items, can be seen as structuring the answers involving the feeling of “Meaningfulness”. The second one, explaining 10.5% of the total variance of the items, seems to capture a dimension of being “Within Some Greater”. The third principal component, explaining 3.8% of the total variance seems to capture “Witnessing Outstanding Standard” evaluation, the fourth principal component, explaining 3.5% of the variance, seems to capture the perception of a “Small Self”, the fifth principal component, explaining 2.27% of the total variance, seem to assemble the items about the “Time Perception”. The sixth principal component, explaining 2.2% of the total variance, seems to capture the dimension of “Mental Challenge” perception. The “Magnitude” item’s specificities as a variable contributing to one singular component is not very clear as it does not reach >.50 loadings on one specific component. The variance of all items is well captured by the extracted components (extracted communality values, *h*^2^ >.5). Also, as mentioned in Study 1, the number of cross-loadings is not surprising, because the cognitive evaluation items were associated to positive emotions and specifically the items were cognitive evaluations specifically related to transcendent emotions. More importantly, to make sense of the extracted dimensions and name them, all the loadings <.32 were ignored.

# Appendix F

**Action tendencies**

## Study 1

*Action tendencies*. Participants were presented with 19 action tendencies and asked to which extent they experienced them in the remembered situation (on a scale from 0 = “Not at all” to 6 = “Very strongly”). The 4 last items were introduced during data collection and were not included in our analysis (*Ns* =2645). All items are presented in Table S15.

Instructions:

“In this situation or just after, to which extent did you feel like performing the following actions or behaviors?” [Scale from 0 (not at all) to 6 (very strongly)]

**Table S15**

*Items for actions tendencies, with their labels and the source they were drawn from.*

| **Item name** | **Question** | **Source** |
| --- | --- | --- |
| 1. *Success1* | Achieving success | Algoe & Haidt (2009) |
| 1. *BetterPerson* | Becoming a better person | Algoe & Haidt (2009) |
| 1. *NiceThings* | Doing random, nice, considerate things for other people | Algoe & Haidt (2009) |
| 1. *ExtraNice* | Doing something extra-nice to someone | Algoe & Haidt (2009)  Zickfeld et al. (2019) |
| 1. *Emulating* | Emulating another person's actions | Algoe & Haidt (2009) |
| 1. *Success2* | Engaging in activities that would lead to professional or academic success | Algoe & Haidt (2009) |
| 1. *PhysicalActivities* | Engaging in physical activities | Algoe & Haidt (2009) |
| 1. *HelpingOthers* | Helping others | Bartlett & DeSteno (2006) |
| 1. *Hugging* | Hugging someone | Zickfeld et al. (2019) |
| 1. *Laughing* | Laughing | Algoe & Haidt (2009) |
| 1. *Learning* | Learning new things |  |
| 1. *MeetingNewPeople* | Meeting new people | Algoe & Haidt (2009) |
| 1. *Smiling* | Smiling | Algoe & Haidt (2009) |
| 1. *SpendingTimeLove* | Spending time with the people I love | Landmann et al. (2019) |
| 1. *CareExpression* | Telling someone how much I care about them. | Zickfeld et al. (2019) |
| 1. *TimeClose* | Spending more time helping people close to me. |  |
| 1. *TimeStrangers* | Spending more time helping people I don't know. |  |
| 1. *HelpingClose* | Helping people I feel close to. |  |
| 1. *HelpingStrangers* | Helping people who are very different from me. |  |

A Principal Component Analysis with Varimax Rotation was conducted on the 15 items measuring the action tendencies. The number of retained components following the Kaiser’s method was three and the Scree Plot method seems to agree with this number. The total variance explained by the three components is 67%.

**Table S16**

*Factor loadings in the rotated component matrix of Principal Component Analysis on items of action tendencies. Bold: loadings >.50*

|  | Component | | |
| --- | --- | --- | --- |
|  | 1 | 2 | 3 |
| 1. Care Expression | **.791** |  |  |
| 1. Extra Nice | **.784** | .345 |  |
| 1. Helping Others | **.752** | .431 |  |
| 1. Nice Things | **.743** | .406 |  |
| 1. Spending Time Love | **.714** |  | .414 |
| 1. Hugging | **.700** |  | .428 |
| 1. Better Person | **.662** | .485 |  |
| 1. Success 2 |  | **.812** |  |
| 1. Learning |  | **.771** |  |
| 1. Success 1 |  | **.761** |  |
| 1. Meeting New People | .321 | **.638** |  |
| 1. Physical Activities |  | **.624** |  |
| 1. Emulating | .414 | **.517** |  |
| 1. Laughing |  | .328 | **.807** |
| 1. Smiling |  |  | **.771** |

The first component, explaining 28.4% of the total variance, seems to capture the dimension of “Prosocial” action tendencies. The second component explains 25.5% of the total variance and seems to capture the “Self-Enhancing” action tendencies, while the third, explaining 13% of the total variance, captures the intention to “Enjoy”. The item “Emulating” was poorly explained by the components (*h*^2^ <.5). Five items are cross-loading (loadings >.40) between the first general component and another dimension, but without invalidating the interpretation of the dimensions (Table S16).

## Study 2

*Action tendencies*. Participants were presented with 27 action tendencies and asked to which extent they experienced while watching the video-clip (on a scale from 0 = “Not at all” to 6 = “Very strongly”). All items are presented in Table S17.

All measures used in Study 1 were also included in this study except for the item measuring the motivation in “Emulating” which was dropped from our questionnaire because the results of Study 1 showed that it didn’t load on any dimension specifically and it was poorly captured by the three components (*h*^2^ <.5).

In addition to items from Study 1, we asked participants to which extent they felt like “helping people in need”, “helping the environment”, “supporting artists”, and “supporting scientists” (on a scale from 0 = “Not at all”, to 6 = “Very much”) as a measure for their intentions to help and as intentions to learn something new.

Instructions:

“While watching the video or just after, to which extent did you feel like performing the following actions or behaviors?” [Scale from 0 (not at all) to 6 (very strongly)]

**Table S17**

*Items for actions tendencies, with their labels and the source they were drawn from (Study 2). Bold characters are for items that has been added in this study and were not present in Study 1.*

| **Item name** | **Question** | **Source** |
| --- | --- | --- |
| 1. *Success1* | Achieving success | Algoe & Haidt (2009) |
| 1. *BetterPerson* | Becoming a better person | Algoe & Haidt (2009) |
| 1. *NiceThings* | Doing random, nice, considerate things for other people | Algoe & Haidt (2009) |
| 1. *ExtraNice* | Doing something extra-nice to someone | Algoe & Haidt (2009)  Zickfeld et al. (2019) |
| 1. *Success2* | Engaging in activities that would lead to professional or academic success | Algoe & Haidt (2009) |
| 1. *PhysicalActivities* | Engaging in physical activities | Algoe & Haidt (2009) |
| 1. *HelpingOthers* | Helping others | Bartlett & DeSteno (2006) |
| 1. *Hugging* | Hugging someone | Zickfeld et al. (2019) |
| 1. *Laughing* | Laughing | Algoe & Haidt (2009) |
| 1. *Learning* | Learning new things |  |
| 1. *MeetingNewPeople* | Meeting new people | Algoe & Haidt (2009) |
| 1. *Smiling* | Smiling | Algoe & Haidt (2009) |
| 1. *SpendingTimeLove* | Spending time with the people I love | Landmann et al. (2019) |
| 1. *CareExpression* | Telling someone how much I care about them. | Zickfeld et al. (2019) |
| 1. ***TimeClose*** | Spending more time helping people close to me. |  |
| 1. ***TimeStrangers*** | Spending more time helping people I don't know. |  |
| 1. ***HelpingClose*** | Helping people I feel close to. |  |
| 1. ***HelpingStrangers*** | Helping people who are very different from me. |  |
| 1. ***Museums*** | Visiting art and/or science museums. |  |
| 1. ***Discovering*** | Discovering new things. |  |
| 1. ***Creative*** | Creating things/being creative. |  |
| 1. ***Understanding*** | Better understanding the world. |  |
| 1. ***Environment*** | Helping the environment. |  |
| 1. ***Artists*** | Supporting artists. |  |
| 1. ***Scientists*** | Supporting scientists. |  |
| 1. ***Improving*** | Improving myself |  |
| 1. ***Need*** | Helping people in need |  |

A Principal Component Analysis with Varimax Rotation was conducted on the 27 items measuring the action tendencies. The number of retained components following the Kaiser’s method was two and the Scree Plot method seems to agree with that choice. The solution in two dimensions can explain 73.8% of the total variance. Nevertheless, the solution in two dimension seems parsimonious but not enough fine-grained for our purposes of distinguishing action tendencies associated to positive emotions. We forced the analysis to retain three, four and five dimensions to see which one gave us the cleanest factor structure and allowed us to investigate more nuances of action tendencies. The solution in four dimensions, explaining 80% of the total variance, appeared to reproduce the three dimensions that were found in Study 1 and to have the least number of cross-loading items and the most interpretable solution (Costello & Osborne, 2005).

**Table S18**

*Factor loadings in the rotated component matrix of Principal Component Analysis on items of action tendencies. Bold: loadings >.50*

|  | Component | |  |  |
| --- | --- | --- | --- | --- |
|  | 1 | 2 | 3 | 4 |
| 1. HelpingClose | **.850** |  |  |  |
| 1. HelpingOthers | **.842** |  |  |  |
| 1. TimeClose | **.837** |  |  |  |
| 1. ExtraNice | **.836** |  |  |  |
| 1. Need | **.833** |  |  |  |
| 1. NiceThings | **.832** |  |  |  |
| 1. SpendingTimeLove | **.803** |  |  |  |
| 1. CareExpression | **.791** |  |  |  |
| 1. HelpingStrangers | **.779** | .347 |  |  |
| 1. BetterPerson | **.778** |  | .369 |  |
| 1. TimeStrangers | **.775** |  |  |  |
| 1. Hugging | **.724** |  |  | .357 |
| 1. Improving | **.677** | .334 | .493 |  |
| 1. MeetingNewPeople | **.554** | .393 | .405 |  |
| 1. Scientists |  | **.810** |  |  |
| 1. Environment | .398 | **.750** |  |  |
| 1. Museums |  | **.730** | .325 |  |
| 1. Discovering |  | **.711** | .387 |  |
| 1. Understanding | .507 | **.689** |  |  |
| 1. Learning |  | **.683** | .461 |  |
| 1. Artists | .343 | **.550** | .409 | .325 |
| 1. Success2 | .381 | .382 | **.720** |  |
| 1. Success1 | .431 | .352 | **.714** |  |
| 1. PhysicalActivities |  | .453 | **.544** |  |
| 1. Creative | .326 | **.519** | **.537** |  |
| 1. Laughing |  |  |  | **.822** |
| 1. Smiling | .454 |  |  | **.724** |

The first component, explaining 37.9% of the total variance, is measured by items describing intentions to positively contribute to relationship with people and seems to capture “Prosocial” action tendencies. The second component, explaining 20.8% of the total variance, is measured by items describing intentions to positively contribute to knowledge and seems to capture “Proknowledge” action tendencies, while the third, explaining 13% of the total variance, captures the “Self-Enhancing” action tendencies, and the fourth component, explaining 8.5% of the total variance, capture the intention to “Enjoy”. Seven items are cross-loading (loadings >.40) between the first general component and another dimension, but without invalidating the interpretation of the dimensions (Table S18).

# Appendix G

**List of variables used in the article**

## Study 1

| Affective States (Emotion Families) | Hedonic State  Social State  Epistemic State  (Antagonist State) *  (Apprehensive State) *  (Negative Self-Evaluation State) *  * not discussed |
| --- | --- |
| Basic Affective Dimensions | Valence  Control  Positive Arousal  Impact |
| Bodily Feelings | Relax  Crying  Shock  Chills  Activation |
| Action Tendencies | Prosocial  Self-Enhancing  Enjoy |
| Cognitive evaluations | Withing Something Greater  Outstanding Standards  Small Self  Situational Valence  Social Connection  Time Perception  Mental Challenge  Optimism About Humanity |

## Study 2

| Affective States (Emotion Families) | Hedonic State  Social State  Epistemic State  (Antagonist State) *  (Apprehensive State) *  (Negative Self-Evaluation State) *  * not discussed |
| --- | --- |
| Bodily Feelings | Excitation  Crying  Relax |
| Action Tendencies | Prosocial  Proknowledge  Self-Enhancing  Enjoy |
| Cognitive evaluations | Meaningfulness  Withing Something Greater  Outstanding Standards  Small Self  Time Perception  Mental Challenge |

# Appendix H

**Communality values for emotional labels**

## Study 1

**Table S19**

*Communality values of emotional labels before and after extraction of six dimensions, representing the variance of each emotional label that is predictable from the extracted dimensions.*

| Communalities |  |  |
| --- | --- | --- |
|  | Initial | Extraction |
| Amusement | 1.000 | .578 |
| Anger | 1.000 | .792 |
| Anxiety | 1.000 | .825 |
| Appreciative | 1.000 | .727 |
| Awe | 1.000 | .397 |
| Compassion | 1.000 | .559 |
| Contentment | 1.000 | .617 |
| Contempt | 1.000 | .467 |
| Disgust | 1.000 | .706 |
| Embarrassed | 1.000 | .737 |
| Enthusiasm | 1.000 | .816 |
| Excitement | 1.000 | .776 |
| Fascination | 1.000 | .718 |
| Fear | 1.000 | .785 |
| Injustice | 1.000 | .703 |
| Grateful | 1.000 | .790 |
| Guilt | 1.000 | .636 |
| Happiness | 1.000 | .871 |
| Hate | 1.000 | .726 |
| Indignation | 1.000 | .659 |
| Inspiration | 1.000 | .644 |
| Interest | 1.000 | .660 |
| Joy | 1.000 | .852 |
| Love | 1.000 | .598 |
| Outrage | 1.000 | .670 |
| Moved | 1.000 | .598 |
| Nervous | 1.000 | .796 |
| Pride | 1.000 | .526 |
| Respect | 1.000 | .587 |
| Sadness | 1.000 | .739 |
| Shame | 1.000 | .790 |
| Surprise | 1.000 | .372 |
| Tenderness | 1.000 | .518 |
| Thankful | 1.000 | .785 |
| Touched | 1.000 | .621 |
| Uplifted | 1.000 | .647 |
| WellBeing | 1.000 | .728 |
| Wonder | 1.000 | .605 |
| Curious | 1.000 | .660 |

## Study 2

**Table S20**

*Communality values of emotional labels before and after extraction of six dimensions, representing the variance of each emotional label that is predictable from the extracted dimensions.*

| **Communalities** | | |
| --- | --- | --- |
|  | Initial | Extraction |
| Admiration | 1.000 | .691 |
| Amusement | 1.000 | .581 |
| Anger | 1.000 | .720 |
| Anxiety | 1.000 | .791 |
| Appreciative | 1.000 | .746 |
| Awe | 1.000 | .696 |
| Compassion | 1.000 | .710 |
| Contentment | 1.000 | .496 |
| Contempt | 1.000 | .513 |
| Curious | 1.000 | .621 |
| Disgust | 1.000 | .583 |
| Embarrassed | 1.000 | .676 |
| Enthusiasm | 1.000 | .706 |
| Excitement | 1.000 | .691 |
| Fascination | 1.000 | .777 |
| Fear | 1.000 | .756 |
| Injustice | 1.000 | .549 |
| Grateful | 1.000 | .742 |
| Guilt | 1.000 | .710 |
| Happiness | 1.000 | .763 |
| Hate | 1.000 | .523 |
| Indignation | 1.000 | .526 |
| Inspiration | 1.000 | .746 |
| Interest | 1.000 | .652 |
| Joy | 1.000 | .765 |
| Love | 1.000 | .740 |
| Outrage | 1.000 | .649 |
| Moved | 1.000 | .783 |
| Nervous | 1.000 | .791 |
| Pride | 1.000 | .554 |
| Respect | 1.000 | .632 |
| Sadness | 1.000 | .629 |
| Shame | 1.000 | .768 |
| Surprise | 1.000 | .456 |
| Tenderness | 1.000 | .735 |
| Thankful | 1.000 | .743 |
| Touched | 1.000 | .831 |
| Uplifted | 1.000 | .760 |
| WellBeing | 1.000 | .647 |
| Wonder | 1.000 | .736 |

# Appendix I

**Other measures**

## Study 1

1. Just after the emotion manipulation (the first open-ended question asking them to describe the emotional situation they just remembered), participants were also asked (i) to describe the feelings and emotions they experienced in this situation, and (ii) to provide five separate words describing these emotions.
2. Participants were asked whether, in the situation they described, their emotion was directed at, or caused by another person. Those who answered YES were presented with 13 more action tendencies and asked to which extent they felt like performing the following actions in the remembered situation or just after.
3. We assessed participants’ disposition to experience various emotions by asking them to complete three different scales: the Geneva Sentimentality Scale (Cova & Boudesseul, 2021), the Dispositional Positive Emotions Scale (Shiota et al., 2006), and the Gratitude Questionnaire-Six Item Form, (McCullough et al., 2002).
4. To investigate the role of emotions in philanthropic behaviour, participants were presented with a list of seven philanthropic foundations and asked how much they would give each foundation if they had $1000 to distribute among them.
5. Finally, participants were asked to provide some information about themselves: age, gender, native language, nationality, number of children, job, education level, education level of their most educated parent if they still were students, religious affiliation, frequency of religious activity and political orientation.

## Study 2

1. Just after watching the video, participants were told that four of them would be drawn at random to receive a £50 bonus, but that they could choose now to give part of this bonus to charities. They were presented with a list of four charities and had to indicate how much of their potential reward they wanted to transfer to each of them.
2. A the end of the questionnaire, we assessed participants’ disposition to experience various emotions by asking them to complete three different scales: the Geneva Sentimentality Scale (Cova & Boudesseul, 2021), the Dispositional Positive Emotions Scale (Shiota et al., 2006), and the Gratitude Questionnaire-Six Item Form, (McCullough et al., 2002).
3. Also, they were asked if they already gave money to charities giving a list of possible non-profit organizations and associations. The possible answers were YES or NO.
4. Finally, participants were asked to provide some information about themselves: age, gender, native language, nationality, number of children, job, education level, education level of their most educated parent if they still were students, religious affiliation, frequency of religious activity and political orientation.

# Appendix J

To determine whether our analysis choices conditioned our results, we ran PCA analyses while including only positive self-transcendent emotions. We found the same structure that we obtained when we included all emotions into the analysis. In Study 1 (see Table S21) we obtain 3 dimensions, including the two same families of self-transcendent emotions: Social states and Epistemic states (the third dimension was composed of self-transcendent emotions like Gratitude and Being thankful, that have a connection to the participants’ self-interest). In Study 2 (see Table S22), we obtained two dimensions of self-transcendent emotion, which corresponded again to Social states and Epistemic states. Thus, changing the analysis method did not change our conclusions regarding the way self-transcendent emotions are structured.

**Table S21**

*Study 1. Factor loadings in the rotated component matrix of Principal Component Analysis on items Self-transcendent Emotions’ labels. Bold: loadings >.50*

|  | Component | | |
| --- | --- | --- | --- |
|  | 1 | 2 | 3 |
| Thankful | **.884** |  |  |
| Grateful | **.883** |  |  |
| Appreciative | **.793** |  |  |
| Inspiration | **.627** | .487 |  |
| Respect | **.621** |  | .380 |
| Love | **.554** |  | **.534** |
| Curious |  | **.796** |  |
| Wonder | .347 | **.721** |  |
| Fascination | .479 | **.710** |  |
| Interest | .387 | **.685** |  |
| Awe |  | **.587** |  |
| Compassion |  |  | **.842** |
| Tenderness | .356 |  | **.666** |
| Touched | .420 |  | **.624** |

**Table S22**

*Study 2. Factor loadings in the rotated component matrix of Principal Component Analysis on items Self-transcendent Emotions’ labels. Bold: loadings >.50*

|  | Component | |
| --- | --- | --- |
|  | 1 | 2 |
| Touched | **.879** |  |
| Tenderness | **.860** |  |
| Love | **.841** |  |
| Compassion | **.840** |  |
| Thankful | **.817** |  |
| Grateful | **.807** | .337 |
| Appreciative | **.743** | .453 |
| Inspiration | **.694** | .494 |
| Admiration | **.663** | **.496** |
| Fascination |  | **.861** |
| Wonder |  | **.812** |
| Curious |  | **.805** |
| Interest |  | **.749** |
| Awe | .409 | **.706** |
| Surprise |  | **.586** |

# References

Algoe, S. B., & Haidt, J. (2009). Witnessing excellence in action: The “other-praising” emotions of elevation, gratitude, and admiration. *Journal of Positive Psychology*, *4*(2), 105–127. https://doi.org/10.1080/17439760802650519

Aquino, K., McFerran, B., & Laven, M. (2011). Moral Identity and the Experience of Moral Elevation in Response to Acts of Uncommon Goodness. *Journal of Personality and Social Psychology*, *100*(4), 703–718. https://doi.org/10.1037/a0022540

Bartlett, M. Y., & DeSteno, D. (2006). Gratitude and prosocial behavior : Helping when it costs you. *Psychological Science*, *17*(4), 319–325. https://doi.org/10.1111/j.1467-9280.2006.01705.x

Costello, A. B., & Osborne, J. W. (2005). Best Practices in Exploratory Factor Analysis : Four Recommendations for Getting the Most From Your Analysis. *Practical Assessment, Research & Education*, *10*, 1–9. https://doi.org/10.7275/jyj1-4868

Cova, F., & Boudesseul, J. (2021). Validation of a measure of participants’ disposition to feel moved: the Geneva Sentimentality Scale. *Submitted Manuscript, University of Geneva*.

Cova, F., Deonna, J., & Sander, D. (2017). “That’s Deep!”: The Role of Being Moved and Feelings of Profundity in the Appreciation of Serious Narratives. In D. R. Wehrs & T. Blake (Eds.), *The Palgrave Handbook of Affect Studies and Textual Criticism* (pp. 347–369). Palgrave Macmillan. https://doi.org/10.1007/978-3-319-63303-9_13

Humbert-Droz, S., Garcia, A. L., Sennwald, V., Teroni, F., Deonna, J., Sander, D., & Cova, F. (2020). Lost in Intensity: Is there an empirical solution to the quasi-emotions debate? *Aesthetic Investigations*, *4*(1), 54–76.

Huta, V., & Ryan, R. M. (2010). Pursuing Pleasure or Virtue: The Differential and Overlapping Well-Being Benefits of Hedonic and Eudaimonic Motives. *Journal of Happiness Studies*, *11*(6), 735–762. https://doi.org/10.1007/s10902-009-9171-4

Landmann, H., Cova, F., & Hess, U. (2019). Being moved by meaningfulness: appraisals of surpassing internal standards elicit being moved by relationships and achievements. *Cognition and Emotion*, *33*(7), 1387–1409. https://doi.org/10.1080/02699931.2019.1567463

McCullough, M. E., Emmons, R. A., & Tsang, J. A. (2002). The grateful disposition: A conceptual and empirical topography. *Journal of Personality and Social Psychology*, *82*(1), 112–127. https://doi.org/10.1037/0022-3514.82.1.112

Piff, P. K., Dietze, P., Feinberg, M., Stancato, D. M., & Keltner, D. (2015). Awe, the small self, and prosocial behavior. *Journal of Personality and Social Psychology*, *108*(6), 883–899. https://doi.org/10.1037/pspi0000018

Saroglou, V., Buxant, C., & Tilquin, J. (2008). Positive emotions as leading to religion and spirituality. *Journal of Positive Psychology*, *3*(3), 165–173. https://doi.org/10.1080/17439760801998737

Schnall, S., Roper, J., & Fessler, D. M. T. (2010). Elevation leads to altruistic behavior. *Psychological Science*, *21*(3), 315–320. https://doi.org/10.1177/0956797609359882

Schnell, T. (2020). *The psychology of meaning in life*. Routledge.

Shiota, M. N., Keltner, D., & John, O. P. (2006). Positive emotion dispositions differentially associated with Big Five personality and attachment style. *Journal of Positive Psychology*, *1*(2), 61–71. https://doi.org/10.1080/17439760500510833

Shiota, M. N., Keltner, D., & Mossman, A. (2007). The nature of awe: Elicitors, appraisals, and effects on self-concept. *Cognition and Emotion*, *21*(5), 944–963. https://doi.org/10.1080/02699930600923668

Silvers, J. A., & Haidt, J. (2008). Moral Elevation Can Induce Nursing. *Emotion*, *8*(2), 291–295. https://doi.org/10.1037/1528-3542.8.2.291

Tabachnick, B. G., & Fidell, L. S. (2013). *Using Multivariate Statistics*. Pearson Education UK.

Yaden, D. B., Kaufman, S. B., Hyde, E., Chirico, A., Gaggioli, A., Zhang, J. W., & Keltner, D. (2019). The development of the Awe Experience Scale (AWE-S): A multifactorial measure for a complex emotion. *Journal of Positive Psychology*, *14*(4), 474–488. https://doi.org/10.1080/17439760.2018.1484940

Zickfeld, J. H., Schubert, T. W., Seibt, B., Blomster, J. K., Arriaga, P., Basabe, N., Blaut, A., Caballero, A., Carrera, P., Dalgar, I., Ding, Y., Dumont, K., Valerie, G., Gracanin, A., Gyenis, R., Hu, C. P., Kardum, I., Lazarevic, L. B., Mathew, L., … Alan, P. F. (2019). Kama Muta: Conceptualizing and Measuring the Experience Often Labelled Being Moved Across 19 Nations and 15 Languages. *Emotion*, *19*(3), 402–424. https://doi.org/10.1037/emo0000450

Zwick, W. R., & Velicer, W. F. (1986). Comparison of Five Rules for Determining the Number of Components to Retain. *Psychological Bulletin*, *99*(3), 432–442. https://doi.org/10.1037/0033-2909.99.3.432
